# Intramolecular Hydrogen Bonding Involving Organic Fluorine: NMR Investigations Corroborated by DFT-Based Theoretical Calculations

**DOI:** 10.3390/molecules22030423

**Published:** 2017-03-07

**Authors:** Sandeep Kumar Mishra, N. Suryaprakash

**Affiliations:** NMR Research Centre, Solid State and Structural Chemistry Unit, Indian Institute of Science, Bangalore 560012, India; sand2epmishra@gmail.com

**Keywords:** NMR spectroscopy, intramolecular hydrogen bond, organic fluorine

## Abstract

The combined utility of many one and two dimensional NMR methodologies and DFT-based theoretical calculations have been exploited to detect the intramolecular hydrogen bond (HB) in number of different organic fluorine-containing derivatives of molecules, viz. benzanilides, hydrazides, imides, benzamides, and diphenyloxamides. The existence of two and three centered hydrogen bonds has been convincingly established in the investigated molecules. The NMR spectral parameters, viz., coupling mediated through hydrogen bond, one-bond NH scalar couplings, physical parameter dependent variation of chemical shifts of NH protons have paved the way for understanding the presence of hydrogen bond involving organic fluorine in all the investigated molecules. The experimental NMR findings are further corroborated by DFT-based theoretical calculations including NCI, QTAIM, MD simulations and NBO analysis. The monitoring of H/D exchange with NMR spectroscopy established the effect of intramolecular HB and the influence of electronegativity of various substituents on the chemical kinetics in the number of organic building blocks. The utility of DQ-SQ technique in determining the information about HB in various fluorine substituted molecules has been convincingly established.

## 1. Introduction

### 1.1. Weak Molecular Interactions

The presence of molecular interactions in Nature cannot be ignored. The existence of various forms of matter, such as, solids and liquids is mainly due to the presence of intermolecular interactions. One can safely make a statement that the world would be a uniform ideal gas in the absence of intermolecular interactions.

The existence of intermolecular interactions is reflected at the molecular level, viz., the thermodynamic non-ideal-gas behavior arising due to vapor pressure, viscosity, virial coefficients, absorption, and superficial tension [1]. The molecular interactions are non-covalent and are inherently electrostatic in nature. These forces could be attractive, repulsive or the combination of attractive as well as repulsive between or within the molecules. They could also be between non-bonded atoms. Intermolecular interactions play predominant role in many fields, such as, conformation of biomolecules, drug design, etc.

**Figure 1:**
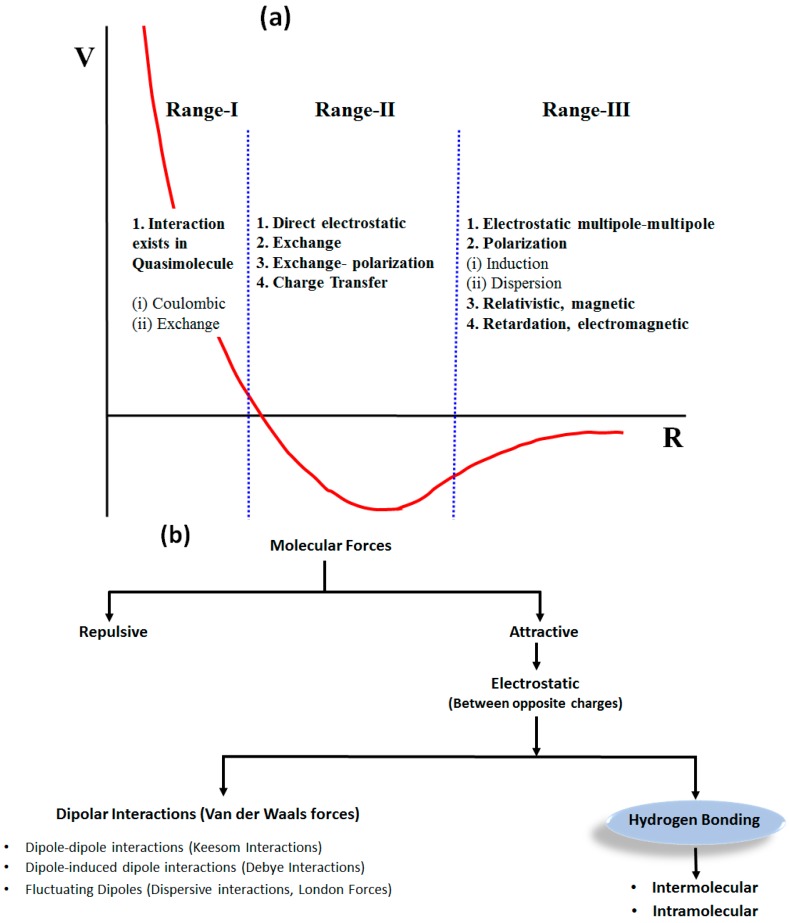
(**a**) The classification of intermolecular interactions on the basis of interatomic distances for a typical interatomic potential. Where *R* is the distance between the centers of masses of the molecules, V is the Lennard-Johns potential and the graph of V as the function of R is called Lennard-Johns model potential graph; (**b**) Schematic illustration of some of known molecular interactions. Some other molecular interactions that are not listed in the scheme may also be possible. This review is mainly focused on the intramolecular HB hence hydrogen bonding is highlighted in the scheme.

Kaplan classified the intermolecular interactions on the basis of distance between the interacting objects [2]. In the classification, there are three main ranges for molecular forces depending on the interatomic distances (*R*) for an interatomic potential (V). For range I the short distances are defined where the potential is repulsive in nature and the electronic exchange is due to the overlapped molecular electronic shells. The range II represents the intermediate distances with the van der Waals minimum, which arises due to the balance between repulsive and attractive forces. Range III represents the large distances with negligible electronic exchange where the intermolecular forces are predominantly attractive. The representation of range of molecular forces is illustrated in Figure 1a. Depending on the nature, the various molecular interactions can also be classified as given in the Figure 1b.

The change in molecular interactions are reflected in physical, chemical and biological phenomenon, such as, phase transitions of water (ice to water to vapor or vice versa), protein folding and unfolding and separation of DNA strands, RNA unfolding, etc. Such processes do not fall under chemical reactions. All the weak molecular interactions have their specific significance and govern the properties of a substrate it may be pointed out that the intermolecular interactions cannot be measured directly by any experiment.

Among all the weak molecular interactions described in Figure 1, except for the hydrogen bond (HB), usually pertain to intermolecular interactions. On the other hand, the HB can be detected within the molecule or between the two or more molecules. This review is focused on the studies carried out by authors’ laboratory on the rare type of intramolecular hydrogen bonding (HB) involving organic fluorine.

### 1.2. Hydrogen Bond

**Figure 2:**
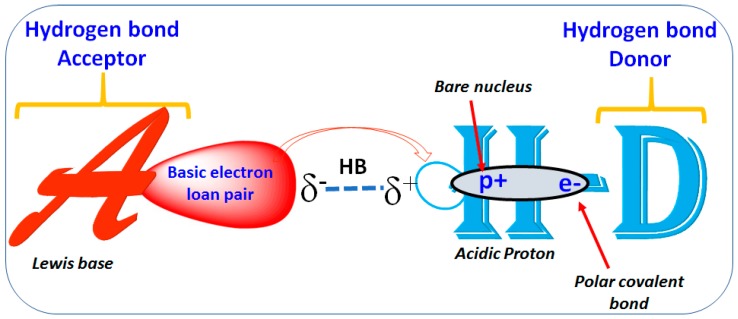
The pictorial illustration of hydrogen bond interaction, where HB acceptor/donor can be F, O, N, or S atom in the molecule. Polarization of electron and exposure of positive proton on either side is shown schematically.

The idea of HB was first suggested by Huggins [3,4,5,6] in 1919, and was further described by Larimer and Rodebush in 1920 [7]. HB is an interaction which occurs between an atom containing a lone pair of electrons (a Lewis base) and a hydrogen atom which is bonded to an electronegative atom (e.g., N, O, S or F) through a covalent bond. In a HB, the Lewis base plays the role of a HB acceptor (A) and the electronegative atom bonded to proton is called the HB donor (D). The hydrogen bonding interaction is schematically depicted in Figure 2.

Hydrogen is the only atom that easily participate in the HB. This is due to the fact that hydrogen atom can form covalent sigma bonds with electronegative atoms like F, N, O and S, etc., where the electron of 1s shells participates in the covalent bond. Since the more electronegative atom has tendency to pull the shared electron pair towards it, in the covalent bond between hydrogen and electronegative atom the bonding electrons moves away from hydrogen nucleus. Due to this the positive charge gets exposed from the back side (the opposite side to the bonded partner). This exposed positive nucleus attracts the partial negative charge of lone pair of the other nucleus. On the other hand, the atoms other than proton having non-bonding electrons in the inner shell shields the nucleus precluding it from getting exposed in this manner.

#### Importance of Hydrogen Bond

Hydrogen bonding is an important non-covalent interaction encountered most often in chemistry and biology [8,9,10,11]. Voluminous information is available on the self-assembly of molecules driven by HB [12,13,14,15,16,17,18,19,20,21,22,23,24]. HB interaction is responsible for the three-dimensional conformation of proteins, synthetic foldamers [25,26,27,28,29,30,31,32,33], peptide catalysis, peptidomimetic design [34,35,36,37,38,39], double helical structure of DNA [40,41], unique properties of water [42], etc. Thus, in-depth knowledge of HB interaction aids in understanding the chemical properties of many organic and bio-macromolecules [43,44,45,46,47,48,49,50,51,52,53].

### 1.3. Organic Fluorine in the HB

A large fraction of the information available in the literature on intramolecular HB pertains to the motifs of the type O···H-N and N···H-N [54,55,56,57,58]. It is well known that organic fluorine hardly ever accepts HB. Dunitz and co-workers have even published a paper entitled “organic fluorine hardly ever accepts hydrogen bonds” [59,60,61,62]. Nevertheless, a few reports are available on the participation of organic fluorine in the HB in the solution state [63,64]. In foldamers and benzanilides, NMR and X-ray crystallographic studies have also been reported on the N-H···F-C HB [65,66]. Recently DFT theoretical studies have been carried out on organofluorine systems and through space J couplings have been also reported [67,68]. The first NMR spectroscopic observation of through space (^1h^*J*_FH_) coupling revealed the information about the involvement of organic fluorine in the HB in the solution state [69]. The Limbach group have reported numerous examples where not only organic fluorine but other halogens also participate in the intermolecular HB [70,71,72,73]. Subsequently this field of research has seen phenomenal growth. Furthermore, the utility of organofluorine molecules as drugs, agrochemicals, biomaterials and also in molecular imaging is well documented [74,75]. Organic fluorine also has enormous importance in molecular association applications, such as crystal engineering [76,77,78] and in the design of the functional materials [79]. Consequent to the binding nature of fluorinated molecules with enzyme active sites, intermolecular hydrogen bonds of the type X-H···F-C (X = O, N) are highly significant, especially in bio-inorganic and medicinal chemistry [80,81,82,83,84]. Despite all its importance in various branches of chemistry and biology, there is limited exploration of organic fluorine-involved HBs, attributed mainly to the fact flourine hardly ever accepts intramolecular HB [85,86,87,88,89]. The present review summarizes the work carried out by authors’ laboratory in recent years, taken directly from their published work.

### 1.4. NMR Spectroscopic Techniques for the Detection of Hydrogen Bond

NMR spectroscopy has proven as a powerful tool in deriving information about HB. Monitoring of the chemical shift as a function of variation in the physical parameters, such as, dilution, temperature is a general technique adopted for gathering information about the HB. The other important parameter that can provide the unambiguous evidence about the existence of HB is through space coupling between two NMR active nuclei mediated by HB. Many of these parameters can be measured by various one and two dimensional NMR experiments.

### 1.5. Theoretical Calculations

The information derived by NMR spectroscopic analysis will conclusively evident if it is also corroborated by theoretical calculations. For such a purpose, various DFT-based theoretical calculations can be carried out. The commonly employed theoretical methods are briefly introduced below.

#### 1.5.1. Non-Covalent Interaction (NCI) Calculations

For the detection of non-covalent interactions in real space, which is dependent on the electron density and its derivatives, NCI [90] calculations are performed. They provide a strong representation of the steric repulsion, van der Waals interactions and the HBs. The calculations yield a large positive gradient of the reduced density gradient (RDG), and the RDG values will be small and approach to zero in the density tail (i.e., regions far from the molecule, where the electron density exponentially decays to zero). This is the condition for both the covalent and non-covalent bonding regions respectively. There will be a strong correlation of electron density (ρ_(r)_) with the weak interactions in the corresponding regions. The correlations for the HB are negative and positive for the steric effect. On the other hand, for van der Waals interaction the ρ_(r)_ values are always small [90] (near to zero). The calculated grid points are plotted for a defined real space function, sign(λ_2(r)_)ρ_(r)_, as function 1 and reduced density gradient (RDG) as function 2.

#### 1.5.2. Atoms in Molecules (AIM) Calculations

For in depth understanding of the molecular properties influenced by weak HBs the interaction energy must be known. The topology analysis technique has been reported and is cited as “atoms in molecules” (AIM) theory. It is also cited as “the quantum theory of atoms in molecules” (QTAIM) [91,92,93,94,95] which is dependent on the quantum observables (electron density ρ_(r)_ and the energy densities). In topology analysis, the critical points (CPs) are those points where gradient norm of function value is zero (except at infinity). According to the negative eigenvalues of the Hessian matrix of the real space function [91,92,93,94,95] CPs can be of four types. Out of these the (3, −1) CP is called the bond critical point (BCP). The value of the real space function at the BCP has a great significance. For example, the bond strength and bond type respectively are related closely to the value of electron density (ρ_(r)_) and the sign of Laplacian of electron density (∇^2^ρ_(r)_) at BCPs [91,92,93,94,95]. The sign of (∇^2^ρ_(r)_) at BCP is important in discriminating shared-shell (covalent bond (−ve)) and closed-shell (ionic, van der Waals interaction, and HB (+ve)). AIM calculations yield the magnitudes of ρ_(r)_ and signs of ∇^2^ρ_(__r__)_ for BCP of HBs of interest. At corresponding (3, −1) critical points (r_cp_) the gradients of electron density (ρ_(r)_) gets vanished. The energy of HB (*E*_HB_) is directly related to potential energy density (*V*_(**r**)_) by straightforward relationship *E*_HB_ = *V*(r_bcp_)/2 [96]. The E_HB_ of X∙∙∙HX type HB can be calculated.

#### 1.5.3. Natural Bond Orbitals (NBO)

Per-Olov Löwdin introduced the concept of *natural orbitals* in 1955, to describe the unique set of orthonormal 1-electron functions which are intrinsic to the *N*-electron wavefunction. NBO is calculated where the electron density could be the maximum. The general sequence of localized orbital is: (1) natural atomic orbitals (NAO); (2) natural hybrid orbitals (NHO); (3) natural bonding orbitals (NBO) and (4) natural (semi-) localized molecular orbitals (NLMO). These localized sets are considered as intermediate between basic atomic orbitals (AO) and molecular orbitals (MO). For the calculation of the electron density distribution in individual atoms and in bonds formed between atoms, NBOs are frequently utilized in computational chemistry. NBOs provide the percentage of highest possible electron density and most possibility for “natural Lewis structure” of ψ. During the formation of HB, the electron transfer from the HB acceptor (lone pairs (lp)) to the anti-bonding orbital (σ*) of the H atom takes place. NBO analysis is proved to be a powerful technique to obtain the detailed information of lp transfer.

#### 1.5.4. Molecular Dynamics (MD)

The idea of molecular dynamics (MD) was first proposed in the 1950s [97]. MD is a computational method for studying the motion of atoms, groups, or molecules. MD studies give dynamic information about the atoms or group of atoms or molecules that interact for a period of time. During MD simulations, mostly the trajectories of atoms/molecules are stabilized where forces and potential energies among the particles are calculated by molecular mechanics force fields or interatomic potentials. MD is also called statistical mechanics by numbers and Laplace’s vision of Newtonian mechanics of predicting the dynamics by animating nature's forces and revealing the information of molecular motion at an atomic scale. MD simulations are also found be very powerful in the detection and quantification of the percentage of different conformers of a molecule where there exists a freely rotating group.

## 2. Studies on Benzanilides

Isomers of fluorinated benzanilides belong to the class of molecules where the cooperative interplay of weak interactions is highly significant in building their higher analogues, called driven foldamers, supra-molecular clusters and bulk moieties such as dendrimers [86,98,99]. Hence these molecules have drawn great attention in structural chemistry. Furthermore, the C-F bond is longer, has opposite bond polarity, stronger than C-H bond and makes the molecule more resistant towards metabolic degradation. As a consequence, the families of these molecules have important biological applications as voltage dependent potassium channel openers [100,101,102,103]. Especially while modeling the higher analogs of benzanilides, three centered N-H···F HBs provide a new approach for the formation of structural features via strong binding effects and induced chirality upon complexation with chiral L-tyrosine-derived ammonium ions [86]. Hence extensive studies have been carried out on the intramolecular HB interactions in the fluorine-substituted derivatives of benzanilides [104].

### 2.1. NMR Spectroscopic Experimental Findings

**Figure 3:**
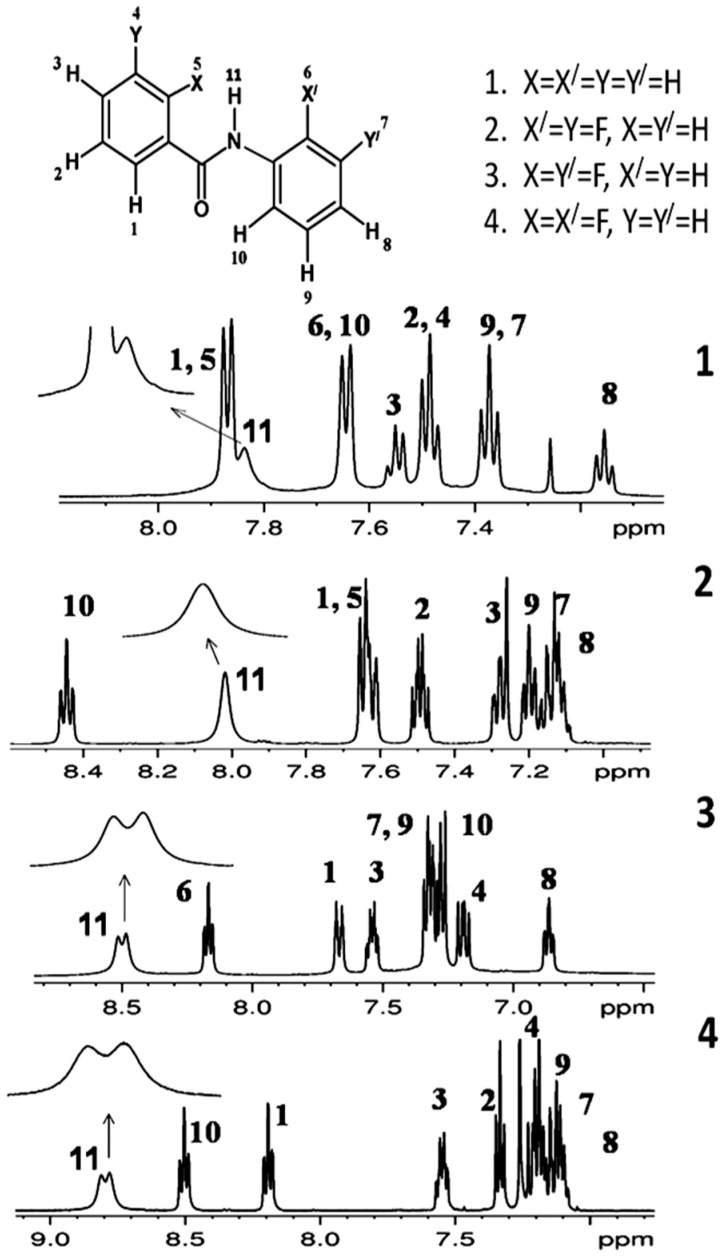
Chemical structures with numbering of interacting spins of fluorine-substituted benzanilides. The 400 MHz ^1^H-NMR spectra of molecules **1**, **2**, **3** and **4** respectively (top trace to bottom trace) in the solvent CDCl_3_. The peak assignments have been made based on multiple quantum (MQ)-single quantum (SQ) correlation experiments. The expansions for amide protons in each molecule are depicted by arrows (reproduced from [103]).

NMR spectroscopy can be used to distinguish between labile free and hydrogen bonded protons, possessing different exchange rates, even in complex molecules if their exchange rates are within the NMR time scale [105,106,107,108,109,110]. Hence information about hydrogen bonding can be derived from the NMR spectral parameters [70,72,111,112,113,114]. Investigations have thus been carried out on a set of four derivatives of fluorinated benzanilides, whose chemical structures are reported in Figure 3, where there the fluorine substitution at positions X and/or X^/^ has been systematically altered.

Unambiguous assignment of aromatic ^1^H resonances have also been made employing the higher quantum correlation experiments for filtering of spectrum corresponding to different spin systems [115,116,117]. The systematic study by employing diverse one and two dimensional NMR experiments revealed information about the weak interactions in all the investigated fluorine-substituted derivatives of benzanilides at ambient conditions.

#### 2.1.1. Detection of Spin-Spin Couplings Mediated by Hydrogen Bonds

**Figure 4:**
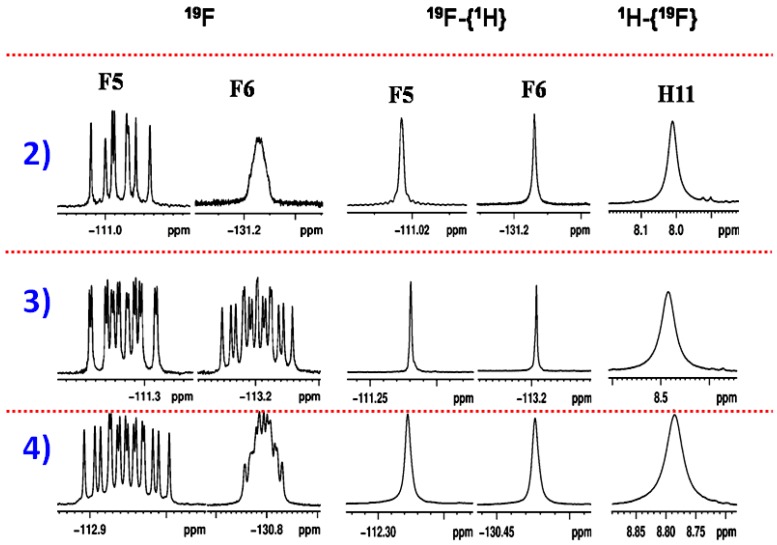
The 400 MHz ^19^F, ^19^F-{^1^H} and ^1^H-{^19^F} NMR spectra of molecules **2**, **3** and **4** in CDCl_3_.

NH proton signals are either singlets (in molecules **1**, **2**) or a partially resolved doublet (in molecules **3** and **4**). The peak separation of the partially resolved doublet is about 16 Hz. These partially resolved doublets might be due to the proton exchange between two distinguishable chemical environments or due to the existence of long range scalar couplings. The particular type of interaction responsible for this can be identified by either two-dimensional exchange spectroscopy (EXSY), or ^1^H-{^19^F} or ^19^F-{^1^H} experiments. The collapse of the NH doublets in both ^1^H-{^19^F} or ^19^F-{^1^H} spectra in the molecules **3** and **4**, is conclusively evident from Figure 4. This unambiguously established that the doublet detected for NH proton is due to the coupling between NH proton and ^19^F. Such a large coupling value of NH proton is very unlikely for ^5^*J*_F(5)H(11)_, ^4^*J*_F(6)H(11)_, ^5^*J*_H(11)H(1)_ and ^4^*J*_H(11)H(10)_ [115,116,117] and has thus been attributed to the coupling between NH proton and ^19^F atom mediated through hydrogen bond bridges between ^19^F and ^1^H.

#### 2.1.2. Simultaneous Detection of Two through Space Couplings

**Figure 5:**
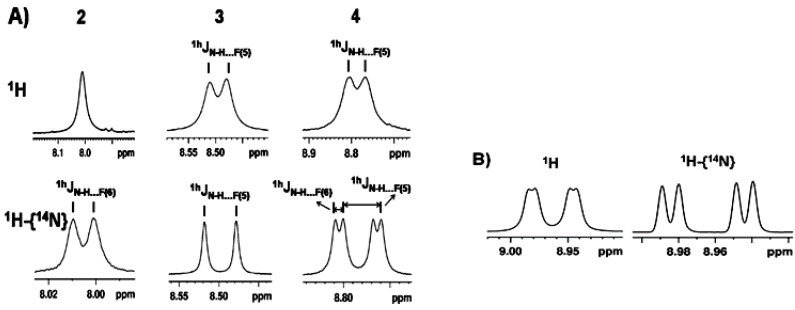
(**A**) The ^1^H (left side) ^1^H-{^14^N} (right side) NMR spectrum of NH proton for the molecules **2**, **3** and **4** respectively at 298 K. (**B**) The ^1^H and ^1^H-{^14^N} NMR spectrum of NH proton for molecule **4** at 230 K.

In the molecules where X and X^/^ are fluorine, the coupling of NH proton with both the fluorine atoms, even if it exists, could not be detected in the one dimensional ^1^H spectrum. The excessively broad NH peak due to quadrupolar ^14^N nucleus prevented the visualization of small couplings, if any. The ^14^N decoupling achieved by systematic variation of the power and frequency of second irradiating RF over a wide range circumvented this problem of quadrupolar broadening and resulted in sharp NH signals as reported in Figure 5A.

Nearly eight-fold reduction in line width of NH peak in **4** (reduced from 16 Hz to 2 Hz), resulted in a distinct doublet of doublet (dd) with measurable couplings of 17.7 Hz (^1h^*J*_N-H_^…^_F(X_^/^_)_) and 3.7 Hz. Similarly, a doublet for molecules **3**, (16 Hz), and a doublet (3.6 Hz pertaining to ^1h^*J*_N-H_^…^_F(X_^/^_)_) for the NH proton of molecule **2** were detected at ambient temperature. Another interesting feature observed was the significant narrowing of NH signal on lowering the temperature (Figure 5B, molecule **4**). This is because of self-decoupling of NH proton with ^14^N, at very low temperatures. However, this fact is not clearly obvious from the ^1^H-{^14^N} spectrum reported in Figure 5B as the decoupling at very low temperatures has very little effect on the line width due to the strengthening of the bifurcated HB at this temperature. Due to the slightly perturbed structural conformation, X^/^(F) is not in close proximity with the amide proton. Thus in this case the hydrogen bond could not be detected by NMR as predicted by single crystal X-ray diffraction studies.

#### 2.1.3. Temperature and Solvent Induced Perturbations

**Figure 6:**
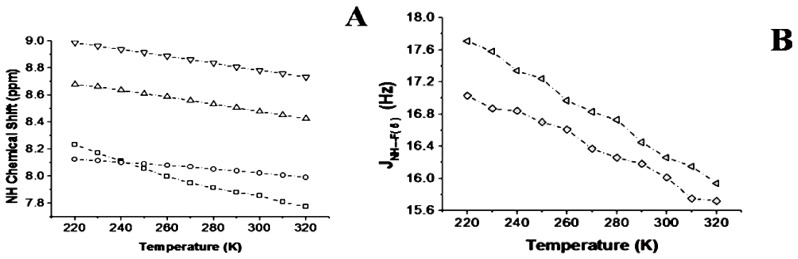
(**A**) Variation of chemical shift of NH protons (δ_NH_) with temperature for all the investigated molecules. The squares, circles, upper triangles and inverted triangles correspond to molecules **1**, **2**, **3** and **4** respectively; (**B**) The variation of ^1h^*J*_N-H...F(5)_, with temperature for molecules **3** and **4**. Squares pertain to molecules **3** and triangles correspond to molecule **4**.

The investigated molecules were subjected to temperature variation over the range of 320–220 K in the CDCl_3_ solvent. The chemical shift of NH protons (δ_NH_) and ^1h^*J*_NH_^…^_F_ were monitored and their dependency on the temperature is plotted in Figure 6A for all the investigated molecules.

HB gets strengthened on lowering the temperature and hence results in deshielding in the ^1^H-NMR peaks as reported in Figure 6A. Another interesting observation is the change in ^1h^*J*_N-H…F(X)_. It varied from 17.03 (220 K) to 15.72 (320 K) Hz and 17.71 Hz (220 K) to 15.94 Hz (320 K) respectively for molecules **3** and **4**, as reported in Figure 6B, which provides clear and straightforward evidence in the favor of HB.

### 2.2. Theoretical Calculations

**Figure 7:**
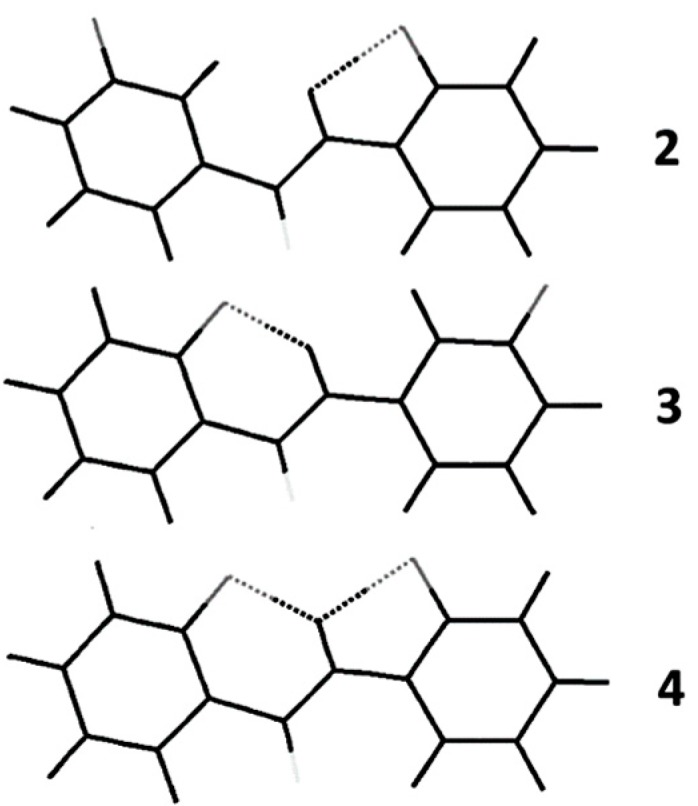
Optimized structures of fluorinated benzanilides visualized using Molden-4.7: HB parameters were measured by their optimization (Molden Control) in the vicinity of F^…^H-N HB distances and angles.

For the fluorinated oligomers ground state geometry optimizations, have been carried out. The geometry optimization was performed to enumerate the intramolecular N-H^…^F distances by employing the B3LYP/6-311+G** basis set [118]. Self-Consistent Isodensity Polarized Continuum Model (SCI-PCM) has been utilized to create the uniform CHCl_3_ solvent environment [119]. Optimized molecular geometries along with HBs are reported in Figure 7.

The theoretically observed HB distances involving N-H^…^F(X^/^) are in close agreement with the X-ray structures [104].

## 3. CF_3_ Substituted Benzanilides

**Figure 8:**
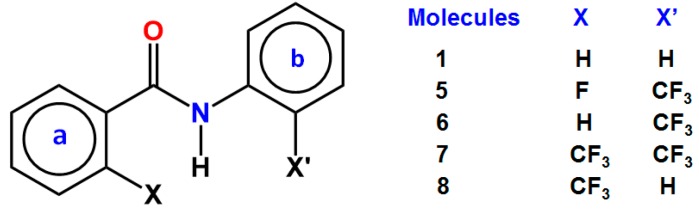
Chemical structure of benzanilide (**1**) and its trifluoromethyl derivatives **5**–**8**.

Extending the study another series of CF_3_ substituted benzanilides have been investigated [120]. The chemical structures of the investigated molecules are reported in Figure 8. In this series of molecules, the unambiguous evidence for the engagement of CF_3_ group in N-H···F-C type HB, in the different CF_3_ substituted derivatives has been observed in a low polarity solvent CDCl_3_. It is well known that C(Sp^3^)-F fluorine is a better acceptor than C(Sp^2^)-F and C(Sp)-F fluorine [121]. To understand the role of CF_3_ group in HB and in structural chemistry an extensive utility of NMR experimental techniques, including variable temperature and dilution studies, has been made. The first example of the engagement of the CF_3_ group in the intramolecular HB of the type C-F···H-N has been reported [121,122,123,124,125,126,127]. The NMR findings have been additionally substantiated by molecular dynamics (MD) simulations.

### 3.1. NMR Experimental Findings

**Figure 9:**
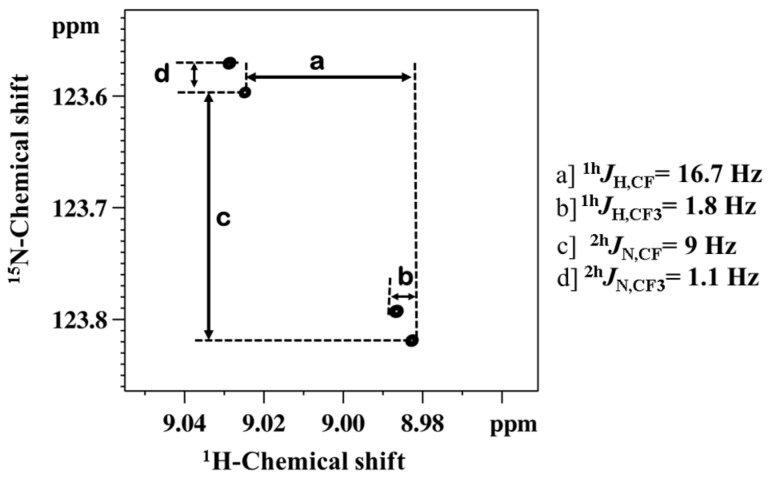
400 MHz two-dimensional ^15^N-^1^H-HSQC spectrum of molecule **5** in the CDCl_3_, depicting the through-space couplings. The measured coupling strengths are identified by a−d and their values have also been reported.

In the ^1^H-NMR spectrum the NH proton of molecule **5** displayed a doublet with a separation of 16.7 Hz [120]. This is attributed to ^1h^*J*_FH_ (coupling mediated through HB) between NH proton and the fluorine of ring a. Collapsing of this doublet to a singlet in the ^1^H{^19^F} experiment unambiguously established that it is mediated through HB, confirming the involvement of F in HB. Analogous to benzanilides discussed in the previous section, even in this molecule the excessive broadening of NH signal was observed, due to quadrupole relaxation by ^14^N, may prevent the precise measurement of small couplings, if any, that may be hidden within the line width. In circumventing such problems, a 2D ^15^N-^1^H HSQC experiment has been carried out where ^15^N is present in its natural abundance. The corresponding spectrum is reported in Figure 9.

The two types of couplings mediated through HB, ^1h^*J*_FH_ and ^2h^*J*_FN_ are reflected in the ^15^N-^1^H-HSQC spectrum. The signs of couplings decided on the basis of the relative slopes of the displacement vectors assuming ^1h^*J*_FH_ to be negative [104].

### 3.2. Theoretical Calculations

**Figure 10:**
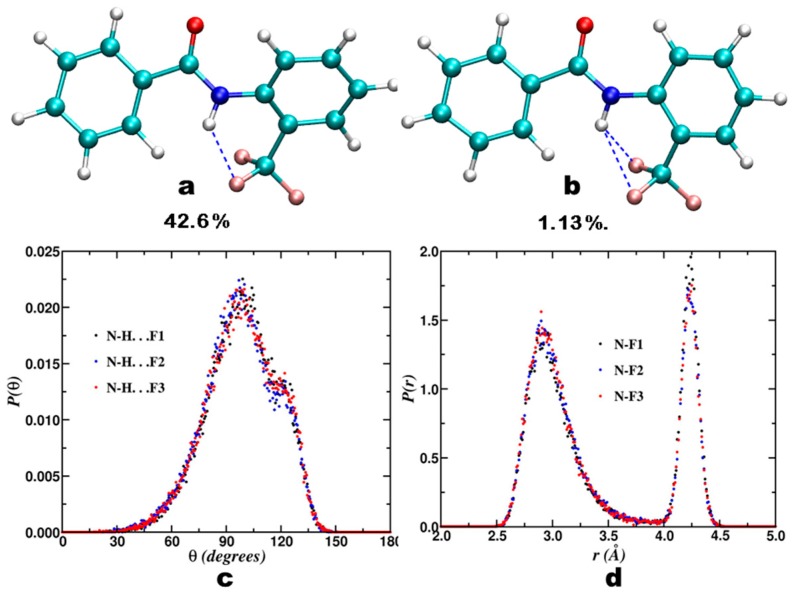
(**a**,**b**) Snapshot of HB formation in molecule **6**, along with their percentage of occurrence, obtained from MD simulations. Blue colored dashed lines show H-bonds; (**c**,**d**) Probability distributions of angles and distances, respectively.

To ascertain the presence of weak molecular interactions established by NMR experiments in these molecules, the DFT based theoretical calculations have been carried out. Gaussian09 has been used at B3LYP/6-311+g (d,p) level of theory [118] for full-geometry optimizations of the molecules **5**–**8**. With the presence of three fluorine atoms in the CF_3_ group there are various possible ways of interactions, such as, two centered, three-centered, or four-centered HBs. To derive the quantitative information about HBs, quantum chemical calculations and MD simulations have been carried out. For calculation, the geometry measurement criteria have been used in which HB is represented as N-H···F, where N is the donor atom and F is the acceptor atom. If the distance, r between N and F atom is less than 3.5 Å and the angle ∠NHF (θ) is greater than 120°, then an HB exists between the N and F atoms. The most probable distance, P(r), and appropriate angle, P(θ), are calculated for all the fluorine atoms in each of these four molecules. The percentage of occurrence of various types of HBs has been calculated from the MD simulations. A snapshot of the formation of HB and their percentage of the occurrence for the molecule **6** is reported in Figure 10.

The calculations reveal that the percentage of occurrence in a situation when there is no HB is 56.27%, whereas the occurrence of a two-centered HB is 42.60% (Figure 10a), and the occurrence of a bifurcated HB is much less and is 1.13% (Figure 10b). Figure 10c,d contain the HB geometrical parameters obtained by MD simulations. It has been observed that all the three F atoms in the CF_3_ group have equal probability of forming HBs, as P(r) and P(θ) are same for three F atoms (Figure 10c,d).

**Figure 11:**
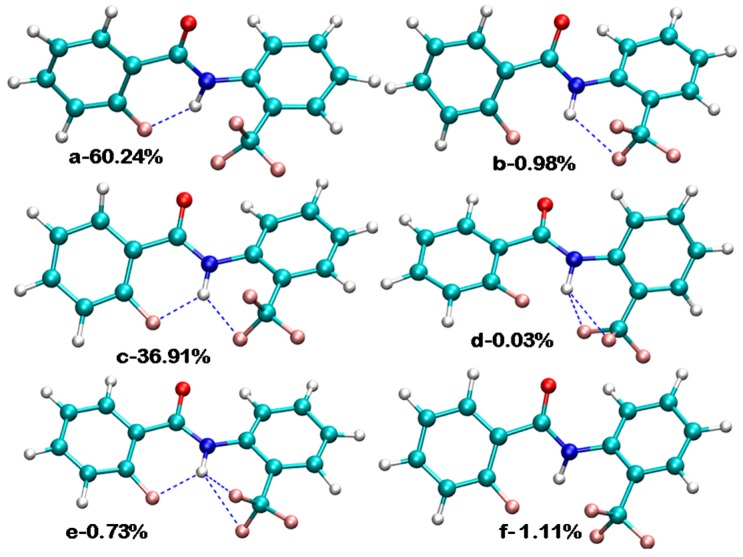
Various possibilities of HB formation for molecule **5**, with their % of occurrence obtained from the MD simulations (HB is shown by blue line).

For molecule **5**, where a single fluorine is substituted on ring “a” and the CF_3_ group on the ring “b”, there are five different possibilities of fluorine getting involved in the HB formation and also a possibility when there is no HB. All such possibilities with their percentage of occurrence determined by MD simulations are reported in Figure 11.

**Figure 12:**
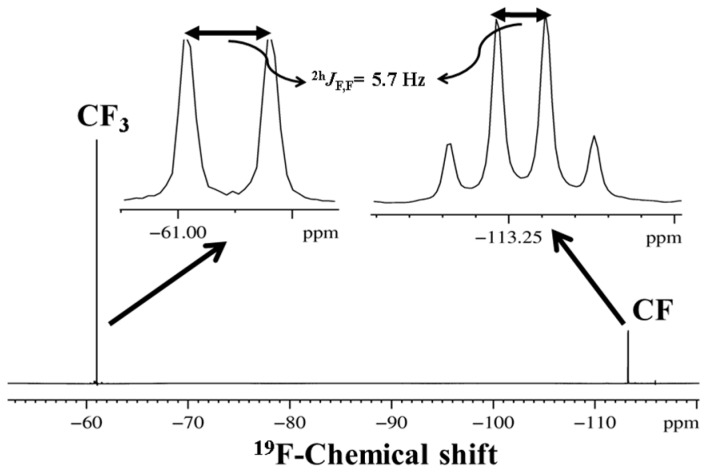
376.7 MHz ^19^F{^1^H} NMR spectrum of molecule **5** showing ^2h^*J*_FF_ through-space coupling.

A very interesting result has been derived from the ^19^F{^1^H} NMR spectrum of this molecule, which exhibited doublet and quartet patterns for CF_3_ and CF groups, respectively. The corresponding spectrum is reported in Figure 12. Fluorine atoms of CF and CF_3_ groups in molecule **5** are separated by eight covalent bonds and the observed doublet peak with separation of 5.7 Hz is quite large. Thus the scalar couplings between the fluorine atoms, mediated through covalent bonds is quite unlikely. It can be safely attributed to the interaction between CF and CF_3_ groups mediated through HB of the type F^…^H^…^F (^2h^*J*_FF_).

**Figure 13:**
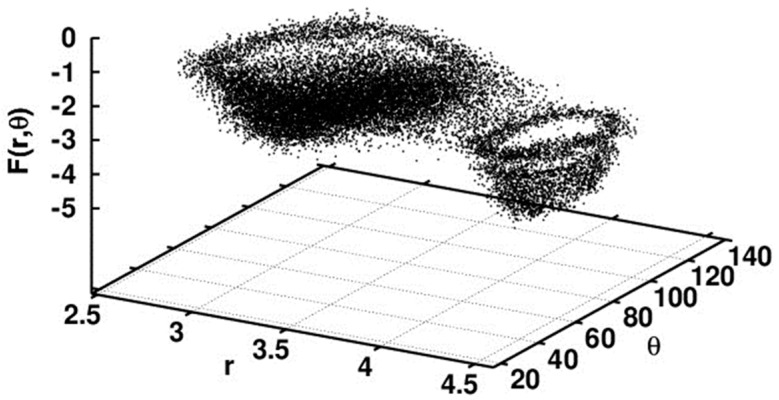
F(r,θ) for N-H···F1-C H-bond occurring in molecule **7**.

The 2D-^19^F-^19^F COSY and *J*-resolved experiments also reflected the cross peaks pertaining to ^2h^*J*_FF_ [120]. The strengths of HB for each fluorine atom present in all the molecules have also been calculated using atomistic molecular dynamics. The histogram of the HB formation H(r,θ) i.e., as a function of the distance, r between donor and acceptor atoms and θ the angle between donor-hydrogen-acceptor atoms, reported in Figure 13, have been calculated at 300 K for molecule **7**, from the 50 ns molecular dynamics simulation trajectory.

The free energy landscape of each atom involved in the HB calculation can be performed from this histogram using formulae given in the Equation (1):

F(r,θ) = −k_B_T ln(2πr^2^sinθH(r, θ)) (1)

For any fluorine atom participating in the HB with N atom, F(r,θ) has two minima corresponding to two states, one with HB and other with no HB. In the free energy landscape for molecule **7** given in Figure 13, for N-H···F_1_-C the minima on the left-hand side is for HB (where r and θ satisfy the conditions for HB) and the other minima belongs to a situation where there is no HB. The strength of HB has also been calculated for the molecules **2**–**5** [120] using Equation (2):

E_hb_ = F^hb^_min_ (r, θ) − F^no−hb^_max_ (r, θ) (2)

where F^hb^_min_ (r, θ) is the minima of hydrogen bonded state and F^no−hb^_max_ (r, θ) is the maxima for no hydrogen bonded state.

## 4. Studies on Hydrazides

Hydrazides are organic compounds that share a common functional group with a N-N covalent bond where at least one of the four substituents should be an acyl group [128]. Different derivatives of hydrazides have proven to be extremely important as, reagents in organic synthesis [129], anti-tumor medicine [130,131], and also in the cytotoxic functioning [132]. In combination with other cmedicines, the derivatives of hydrazides are utilized for the treatment or prevention of tuberculosis [133].

### 4.1. N,N-Diacyl Substituted Derivatives of Hydrazides

The hydrazides provide a possibility to synthesize a variety of derivatives with different combinations of substituted acyl group(s) of interest. The different derivatives of hydrazides have been synthesized and characterized using one and two dimensional multinuclear NMR experiments, and ESI mass spectrometry. The procedure for synthesis of these molecules and the NMR spectral analysis have been reported [134]. The NMR experiments reveal the existence of weak intramolecular interactions in all the investigated derivatives of hydrazides [134]. The NMR derived HB information has been further ascertained by DFT [135,136] based NCI [90], and QTAIM [91,92,93,94,95] calculations.

#### 4.1.1. NMR Spectroscopic Detection

**Figure 14:**
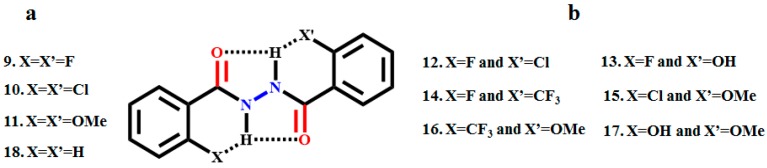
The chemical structures of 2-X-*N*-(2-X’) benzohydrazide derivatives; (**a**) symmetrically substituted molecules and (**b**) asymmetrically substituted molecules.

One of the NMR spectral parameters that provides information on the HB is the variation of chemical shift under different experimental conditions. In the reported work [134] the different derivatives of hydrazide, **9**–**18**, generically named 2-X-*N*-(2-X’)benzohydrazides, whose basic chemical structures and their site specific substituents, reported in Figure 14, have been investigated. The disubstituted molecules are classified into two categories, one where X = X’ (**9**–**11** and **18**) and the other where X ≠ X’ (**12**–**17**).

**Scheme 1:**
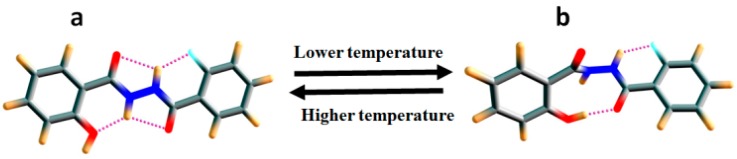
Two different possible stable conformations of molecule **13**, (**a**) at higher temperature; and (**b**) at lower temperature.

**Figure 15:**
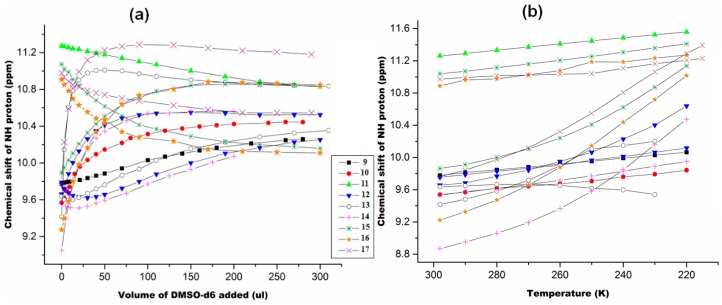
(**a**, **b**) Variation in the chemical shifts of NH proton as a function of temperature and the volume of DMSO-*d*_6_ respectively for the molecules **9**–**17**. The initial concentration was 10 mM in the solvent CDCl_3_. (**a**) The DMSO-*d*_6_ was incrementally added to an initial volume of 450 µL in CDCl_3_, at 298 K; (**b**) The temperature was varied from 300 to 220 K. The molecules **9**–**17** are identified by the symbols given in the inset.

For discarding any possibility of dimerization or self-aggregation, if any, 2D DOSY [137,138] experiments, ESI-MS analysis and dilution studies have been carried out. Solvent titration experiment [63,64,139] has been employed to understand the weak interactions, such as, intra- and inter- molecular HB, to compare their relative strengths of interaction and also to evaluate the effect of monomeric water on HB, which is absorbed from the atmosphere. The variation in the chemical shift of NH proton as a function of temperature (300–220 K) is compared for all the investigated molecules in Figure 15b. On lowering the temperature, the NH peak of all investigated molecules except one NH proton peak of molecule **13** is showed downfield shift due to the strengthening of HB. The unusual behaviour of this NH proton of the molecule **13** has been attributed to the switching of this molecule to another possible conformer on lowering the temperature. The possibility of such a switching phenomenon is illustrated in Scheme 1.

The relative strength of intramolecular HBs has been qualitatively derived by titration with dimethylsulphoxide (DMSO) solvent [134]. The observed variation in the chemical shifts as a function of the incremental addition of DMSO-*d*_6_ is plotted for the molecules **9**–**17**, in Figure 15c. Disruption of intramolecular HB [104] by the solvent DMSO results in the deshielding of NH protons. On the contrary for the NH protons of molecule **11**, and one of the two NH protons of asymmetric molecules, **15**–**17** the high field shift was detected on addition of DMSO-*d*_6_. This is due to the fact that these NH protons involved in HB with OMe group are relatively stronger than the DMSO interaction. The high field shift of these protons is attributed to an equilibrium which is stabilized between intra- and inter- molecular hydrogen bonded species.

**Figure 16:**
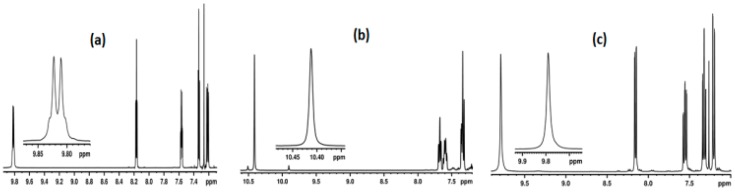
400 MHz ^1^H-NMR spectrum of molecule **9**, (**a**) in CDCl_3_; (**b**) ^1^H-NMR spectrum in DMSO-*d*_6_ and (**c**) ^1^H{^19^F} NMR spectrum in CDCl_3_.

Another strong evidence in the favour of organic fluorine involved intramolecular HB is the detection of through space couplings between the NH proton and fluorine [40,140,141,142]. The ^19^F coupled and decoupled ^1^H spectrum of molecule **9** in the solvent CDCl_3_, and the ^19^F coupled spectrum in the solvent DMSO-*d*_6_ are given in Figure 16. The NH peak of the molecule **9** is a doublet with a separation of 12.75 Hz (Figure 16a). This doublet collapsed into a singlet in the ^1^H{^19^F) experiment confirming the presence of the coupling between ^1^H and ^19^F (Figure 16c). The doublet also collapsed to a singlet in a high polarity solvent DMSO-*d*_6_ (Figure 16b).

**Figure 17:**
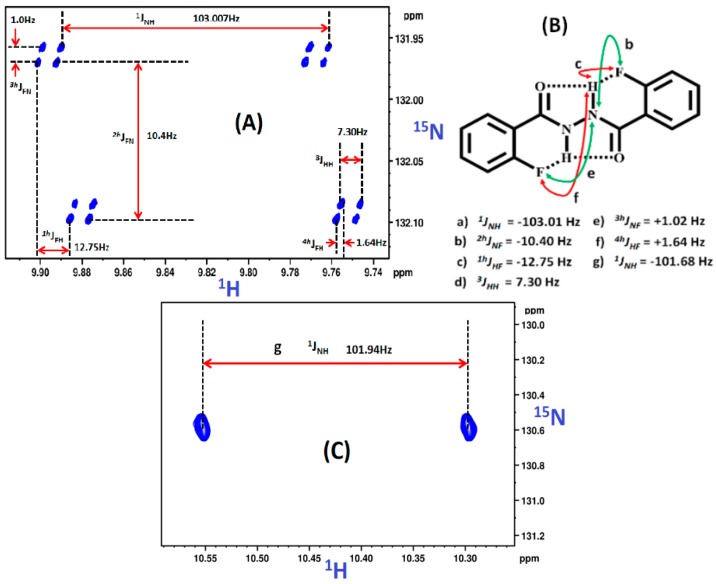
(**A**) 800 MHz ^1^H-^15^N-HSQC (NH-coupled) spectrum of molecule **9** in CDCl_3_; (**B**) the chemical structure of the molecule **9** with marking of couplings determined and their magnitudes; (**C**) 400 MHz ^1^H-^15^N-HSQC spectrum (NH-coupled) in DMSO*-d*_6_.

The ^3^*J*_HH_ and ^4h^*J*_FH_ couplings, if any, are not detectable due to the symmetry of the molecule. For detection of these couplings the symmetry of the molecule has to be broken. For such a purpose 2D ^1^H-^15^N HSQC experiment has been carried out for the molecule **9**, where ^15^N is present in its natural abundance. The HSQC spectrum is reported Figure 17A where all the possible seven couplings, ^1^*J*_NH_, ^2h^*J*_FN_, ^2^*J*_NH_, ^3h^*J*_FN_, ^1h^*J*_FH_, ^4h^*J*_FH_ and ^3^*J*_HH_ has been measured from this spectrum. The observation of through space couplings of significant strengths, such as ^1h^*J*_FH_, ^2h^*J*_FN_^3h^*J*_FN_ and ^4h^*J*_FH_, provided strong and direct evidence for the involvement of organic fluorine in the intramolecular HB. In the solvent DMSO, except for ^1^*J*_NH_, all the other couplings mediated through HB disappeared as reported in Figure 17B.

The relative signs of all the couplings were determined with respect to coupling c marked in Figure 17B. From this it was inferred that ^1h^*J*_FH_ is negative. The comparison of ^1^*J*_NH_ [143,144] of molecules **9**–**17** with unsubstituted molecule **18**, provided ample evidence that the nature of HBs in derivatives of hydrazides are predominantly covalent in nature. The close proximity of NH proton with the fluorine in fluorinated molecules is detected by 2D HOESY (hetero-nuclear Overhauser effect spectroscopy) experiment. This also provided evidence in favor of intramolecular HB.

#### 4.1.2. NCI Plot

**Figure 18:**
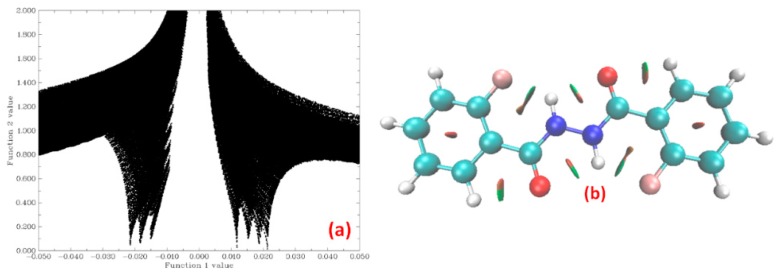
(**a**) The plot of sign(λ_2(r)_)*ρ_(r)_ as function 1 v/s the RDG as function 2; and (**b**) coloured isosurface plot (green color denotes weak H-bond and red color stands for steric effect) for molecule **9**.

The weak molecular interactions established by NMR studies have also been corroborated by theoretical DFT [135,136] optimized structure based calculations. The calculation of noncovalent interaction (NCI) has been shown to be very useful technique for the visualization of weak interactions [90]. The calculated grid points are plotted for a defined real space function, sign(λ_2(r)_)ρ_(r)_, as function 1 and reduced density gradient (RDG) as function 2 and also the color filed isosurfaces for all the investigated molecules. Figure 18 gives these plots for the molecule **9**.

There are three spikes on the left hand side of Figure 18a (i.e., sign(λ_2(r)_)*ρ_(r)_ is negative) denoting three HBs, viz., N-H···F, N-H···O and C-H···O. These three HBs can be observed in Figure 18b as green coloured isosurfaces. The red colour in isosurface plot (Figure 18b) represents the steric hindrance arising from phenyl ring of the molecule and other HB mediated rings. This is seen as four spikes on the right hand side (i.e., sign(λ_2(r)_)*ρ_(r)_ is positive) of Figure 18a. Similar results have been derived for all other investigated molecules [134].

#### 4.1.3. Atoms in Molecules (AIM) Calculations

The magnitudes of ρ_(r)_, signs of ∇^2^ρ_(r)_ and potential energy density (*V*_(**r**)_) at corresponding (3, −1) BCP (bond critical points) (r_cp_) for HBs of interest have been calculated using QTAIM calculations [91,92,93,94,95]. The value of (*V*_(**r**)_) used in the *E*_HB_ = *V*(r_bcp_)/2 [96] for the calculation of energy of HB (*E*_HB_) of X∙∙∙HX type. The calculated *E*_HB_ of different HBs in all investigated molecules ranged between −3 to −8.6 Kcal/mol [134].

The ^1^H-NMR spectra were simulated using the GIAO [145] and CSGT [145] methods. It is observed that in most of the cases there are more than one conformers for the molecules. Thus the calculated chemical shift values of NH protons are not in complete agreement with the experimental data. The values observed from CSGT method are comparable with the experimental results.

### 4.2. N, N Acyl-Phenyl Substituted Derivatives of Hydrazides

**Figure 19:**
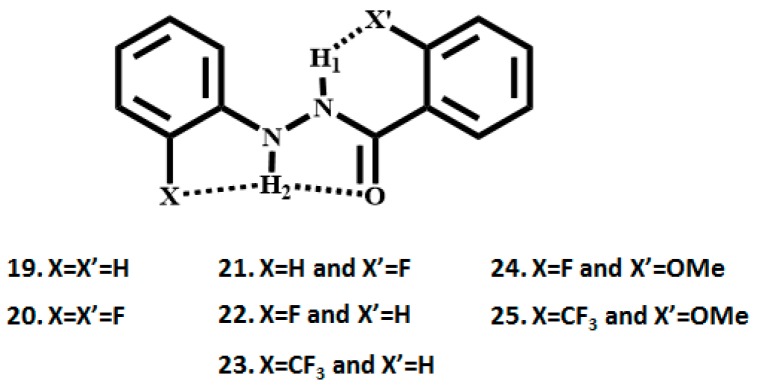
The chemical structures of *N*′-phenylbenzohydrazide and its halo derivatives. The dotted lines indicate the HB.

In this work another series of hydrazide derivatives has also been synthesized and investigated, where the formation of five and six membered rings mediated through HBs is possible. The general chemical structure is given in the Figure 19.

The main focus of this work is to determine the conformations of the fluorinated hydrazide derivatives and thereby understanding the effect of HB of the type F/CF_3_^…^H (N).

#### 4.2.1. Spectroscopic Experimental Observations

**Figure 20:**
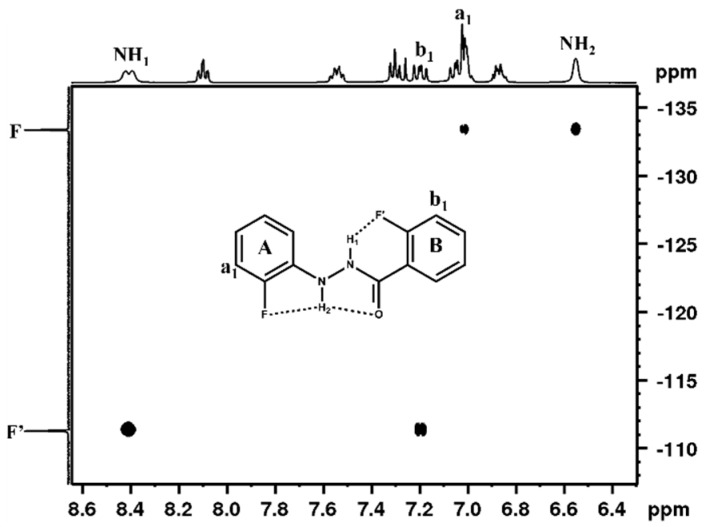
400 MHz two dimensional ^1^H-^19^F HOESY spectrum of molecule **20**. The detection of strong cross peaks convincingly established the correlations between NH_(1)_ proton and F and also NH_(2)_ proton and F. The cross peaks were also observed for phenyl protons a_1_ and b_1_. The mixing time used in the experiment was 450 ms.

For initiating the study, the ^1^H-^19^F HOESY experiments have been carried out for all the investigated molecules and the strong correlation in ^1^H-^19^F HOESY spectrum is observed where fluorine (X’) in the phenyl ring B established correlation with NH_(1)_ proton whereas fluorine in the phenyl ring A (X) established correlation with NH_(2)_ proton. From the obtained correlations, the conformations of all the synthesized molecules have been derived and ^1^H-^19^F HOESY spectrum for the molecule **20** is reported in Figure 20.

After arriving at the conformations of the molecules, the further studies were directed to derive the information about the weak molecular interactions (HB) that validate the proposed conformations. The titration experiment with CDCl_3_ did not show any significant change in the chemical shift of NH protons confirming the absence of any intermolecular interactions. The peak at 1.54 ppm corresponding to monomeric water remain constant indicating the absence of hydrizide-water interaction [146]. For discarding the possibility of dimerization or aggregation, if any, DOSY experiment has been carried out with the mixture of 1:1 molar ratio of molecules **20** and **25** in the solvent CDCl_3_, which have different diffusion coefficients.

**Figure 21:**
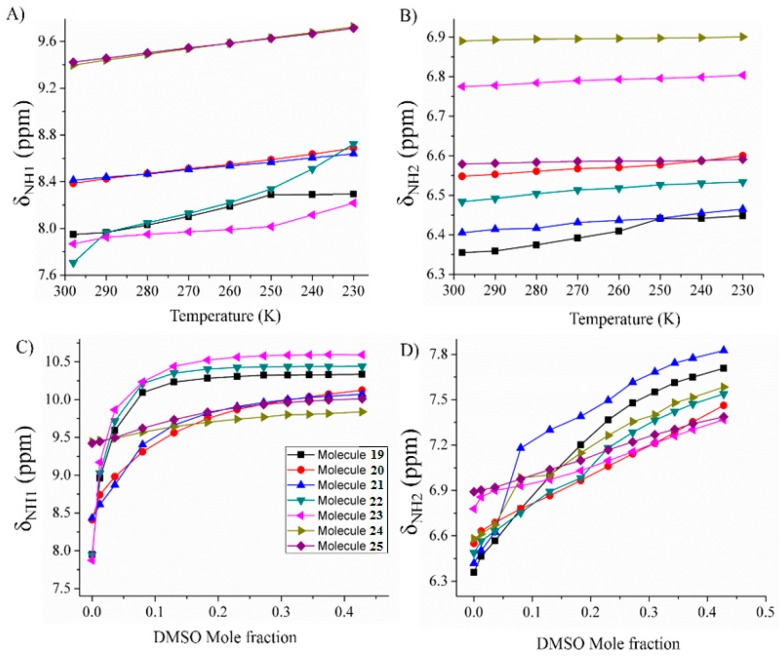
The variation of the chemical shift of; (**A**) NH_(1)_; (**B**) NH_(2)_ protons with temperature; (**C**) NH_(1)_ and (**D**) NH_(2)_ protons with the incremental addition of DMSO-*d*_6_ for the molecules **19**–**25** in the solvent CDCl_3_ at 298 K. The color code for the molecules are given as an inset in Figure 21C, and is same for Figure 21A–D.

The ^1^H-NMR spectra for the investigated molecules have been acquired at different temperatures where the HB gets strengthened on lowering of temperature and results in deshielding of the NH proton participating in HB. The corresponding plots of temperature dependent variation of chemical shifts of NH protons are reported in the Figure 21A,B. In addition to this the incremental additions of DMSO-*d*_6_ resulted in the deshielding of NH proton providing information about the strength of HB. The perturbation on the chemical shifts of NH proton (δ_NH_) induced by DMSO-*d*_6_ are given in Figure 21C,D. The temperature coefficient is directly proportional to the strength of HB. On the other hand, the Δδ_NH_/ΔV_DMSO_ has inverse relation to the strength of HB. Hence mere visual inspection of the graphs given in Figure 21 provides information about the comparative strengths of HB in the studied molecules.

**Figure 22:**
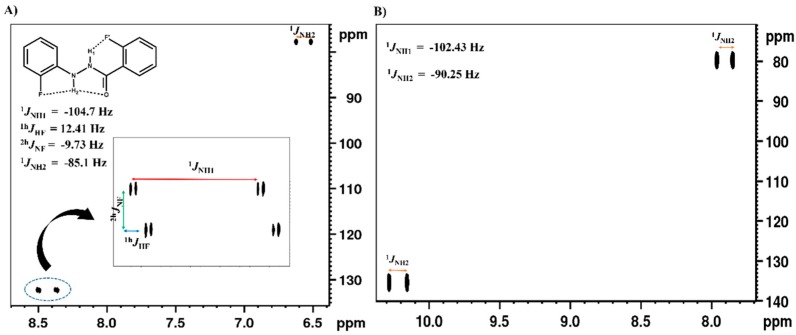
400 MHz coupled ^15^N-^1^H-HSQC spectra of molecule **20** in the solvents (**A**) CDCl_3_ and (**B**) DMSO-*d*_6_. The measured couplings and their signs are reported in the Figure 22A.

The direct evidence about the presence of HB can be obtained by deriving the coupling between two NMR active nuclei which are participating in the formation of HB. In order to overcome the broadening (due to quadrupolar ^14^N nucleus) in the NMR spectrum, the 2D ^15^N-^1^H HSQC spectra have been recorded for all the investigated molecules where ^15^N is in natural abundance. The expanded NH_(2)_ region of ^15^N-^1^H HSQC spectrum of molecule **20** in CDCl_3_ is given in Figure 22 which provides values of ^1h^*J*_FH_ and ^2h^*J*_NF_. These ^1h^*J*_FH_ and ^2h^*J*_NF_ detected in the solvent CDCl_3_, disappeared in the solvent DMSO-*d*_6_. This unequivocally established that these are coupling arising from through space interactions.

Furthermore, in the 2D ^15^N-^1^H HSQC spectrum of molecule **25**, the NH_(2)_ peak appeared as a quartet in CDCl_3_ while it appeared as a singlet in DMSO solvent indicating that the couplings observed in CDCl_3_ are the through space contribution [134]. The ^1^*J*_NH_ scalar coupling is another important NMR parameter for extracting the information about the nature of HB [40,144,147]. There is a significant increase in ^1^*J*_NH(1)_ and ^1^*J*_NH(2)_ values in halo/methoxy substituted molecules compared to the unsubstituted molecule of hydrazide, confirming that the corresponding HBs are predominantly electrostatic in nature.

#### 4.2.2. Theoretical Calculations

The observed NMR experimental results have been corroborated by IR and QTAIM [91,92,93,94,95], NCI [90] and the conformational study by using relaxed potential energy scan. The chemical structures of all the synthesized molecules have been optimized using G09 program with B3LYP/6-311G** level of theory by taking chloroform as a solvation medium. The molecular structures were also optimized with larger basis set like aug-cc-pVTZ and ^1^H-NMR spectra were simulated using GIAO [145] method for optimized structures (both by 6-311G** and aug-cc-pVTZ basis sets) in chloroform solvation medium.

Additional information has been derived by IR and QTAIM an NCI analysis. The C=O and N-H stretching frequencies in the IR spectra of all the investigated molecules showed blue shift, providing an evidence for the existence of HB [148]. Positive sign for Laplacian of electron density (∇^2^ρ) at the NH_(1)_^…^X’ [148] indicates that in all the molecules the type of interaction is HB.

The NCI index detects the weak interactions in real space based on the electron density and its derivatives. Using Multiwfn program, the grid points have been calculated and plotted for the two functions: sign(λ2)*ρ as function 1, and reduced density gradient (RDG) as function 2. Both these functions have the information about HB. The color filled isosurface graphs show the presence of H(N)^…^X–C and C=O^…^H(N)^…^X–C type HBs in the investigated molecules. These calculations further supported the NMR observations.

##### Relaxed Potential Energy Scan

**Figure 23:**
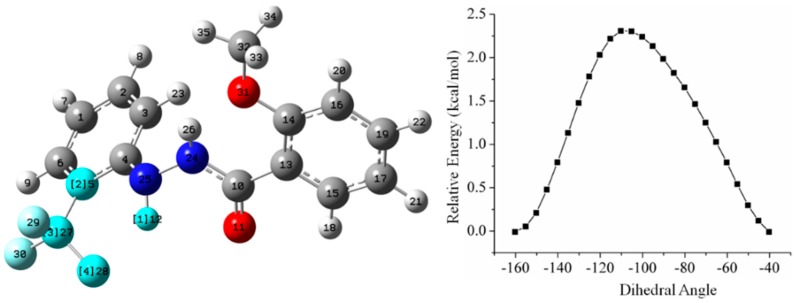
Relaxed potential energy surface Scan for the internal rotation of the CF_3_ group. The scan was performed in 28 steps with increments of 5° in the dihedral angle.

In NMR study, the NH_(2)_ proton of molecule **25** appeared as a quartet in ^1^H-^15^N HSQC spectrum. To understand the reason behind the observation of quartet the relaxed potential energy scan has been performed using B3LYP/6-311G** level of theory at ambient temperature for the F28–C27–C5–H12 dihedral angle through which the internal rotation of the CF_3_ group was confirmed (Figure 23). The calculated energy barrier for the CF_3_ internal rotation is about 2.31 kcal/mol. Because of such a low energy barrier CF_3_ group appeared as a quartet in NMR spectrum.

**Figure 24:**
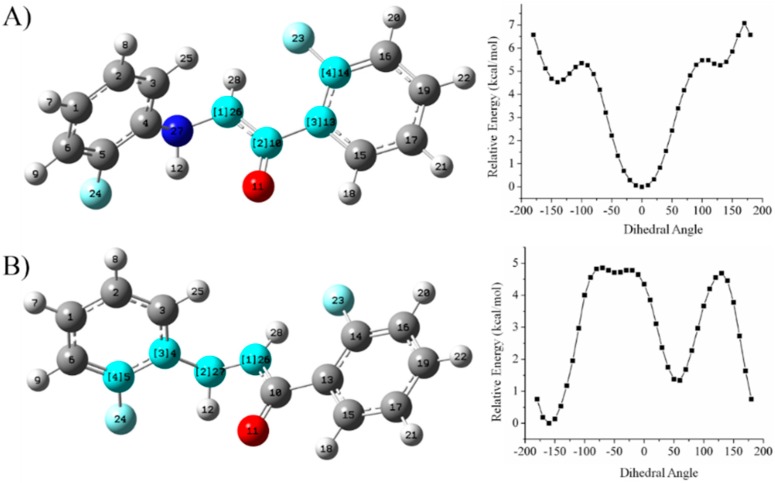
Relaxed potential energy surface scan for the internal rotation of phenyl ring through a single bond in molecule **20**. Selected dihedral angles; (**A**) N26–C10–C13–H14 (**B**) N26–N27–C4–C5.

The relaxed potential energy surface scan has also been carried out for the molecule **20** using B3LYP/6-311G** level of theory at ambient temperature in 36 steps with 10° increment in the dihedral angle (−180° to +180°) in order to get the information about the internal rotation of the phenyl ring through a single bond. From Figure 24 it is clear that the proposed conformation has lower energy over the other.

## 5. Studies on Derivatives of Imides

**Figure 25:**
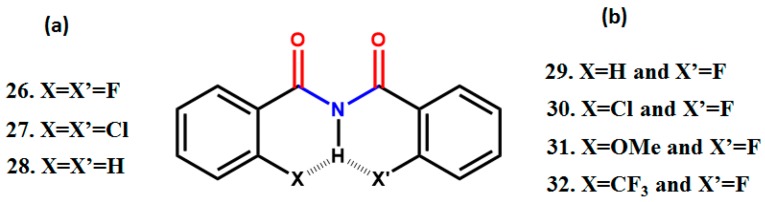
The chemical structures of the derivatives of 2-X-*N*-(2-X’-benzoyl)benzamide.

The imides are the diacyl derivatives of ammonia or the primary amines [128]. The importance of imide derivatives is found in many field of daily interest such as, high strength electrically conductive polymers [149,150,151], synthetic applications [152], medicinal activity [153], as ionic fluids [154], in pharmacology [155] and as synthetic precursors [156] are well documented. The NH linker of an imide provides ample scope to synthesize different derivatives with the desired substitution on acyl group(s). Several derivatives of imides whose chemical structures are given in the Figure 25 have been synthesized by using microwave assisted method [157] and characterized by extensive utility of NMR techniques and ESI-HRMS spectrometry [158].

### 5.1. Information Derived by NMR Spectroscopy

The NMR spectroscopic derived information on intramolecular HB in these molecules has been unequivocally supported by Density Function Theory DFT [135,136] based NCI [90,159], and QTAIM [91,92,93,94,95] calculations.

The solvent titration experiments [62,63,140] have been performed on the molecules **26**–**32** in the solvent CDCl_3_ to distinguish the intra- and inter- molecular HB and to monitor the effect of atmospheric monomeric water on HB. It is observed that there is no change in the chemical shift of NH proton as well as the residual water peak position in the deuterated solvent [158]. These observations discarded any possibility of intermolecular HB, aggregation, dimerization or water-imide interaction.

**Figure 26:**
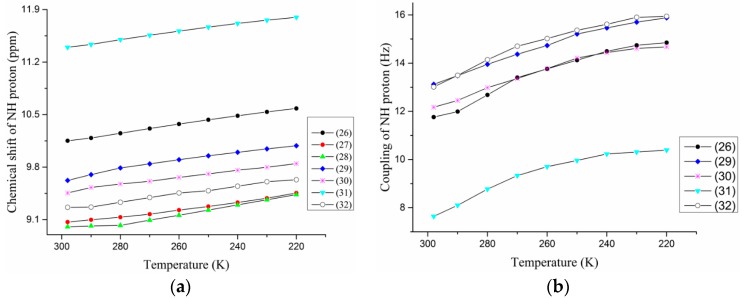
Variation of (**a**) chemical shifts of NH protons as a function of temperature for the molecules **26**–**32** and (**b**) through space mediated HF coupling constant as a function of temperature for the molecules **26** and **29**–**32**. The molecules are identified by the symbols given in the inset. The initial concentration was 10 mM in CDCl_3_.

DMSO-*d*_6_ solvent titration has been employed to derive the qualitative information on the relative strengths of intramolecular HB on the molecules **26**–**32**. The excessive deshielding of NH proton is observed as a consequence of the disruption of intramolecular HB due to the strong interaction with DMSO. The upfield shift for NH peak with the addition of DMSO [158] in the molecule **6**, indicated that in this particular molecule, the intramolecular HB formed between oxygen atom of the methoxy group and NH proton might to be stronger than its interaction with DMSO [134]. The strength of HB gets increased on lowering the temperature. Excessive deshielding of the NH proton is observed on lowering the temperature due to the displacement of hydrogen bonded proton towards the HB acceptor. This is an evidence in favor of intramolecular HB [160,161,162]. The chemical shift of NH protons as a function of temperature (over the range of 298–220 K) for molecules **26**–**32** are contained in Figure 26A.

The change in FH coupling value on lowering the temperature has also been detected. Such a variation is possible only when the spin polarization is transmitted between two NMR active nuclei mediated through HB. The variation in the coupling constant (through space) as a function of temperature is reported in the Figure 26B, for the molecules **26** and **29**–**32**.

The GIAO [145] and CSGT [145], DFT methods of NMR simulation have been used for chemical shifts calculation and it has been observed that the CSGT method is giving the values that are in close agreement with the experimentally observed values.

**Figure 27:**
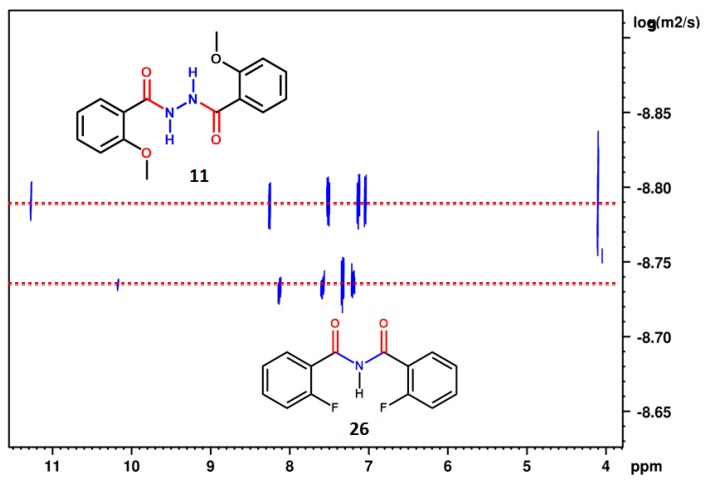
500 MHz ^1^H-DOSY NMR spectrum of 20 mM solution of the mixture of molecules **11** and **26** at a 1:1 molar ratio in CDCl_3_.

It is obvious that the molecule 2-methoxy-*N*’-(2-methoxybenzoyl)benzohydrazide labelled as **11** whose chemical structure is given in Figure 27, does not show any type of self-dimerization or aggregation [134], even in 20 mM solution. The ^1^H-DOSY [137,138] NMR experiment has therefore been carried out for a mixture of 1:1 molar ratio (20 mM final solution) of molecules **11** and **26** in the solvent CDCl_3_ and the corresponding spectrum is reported in Figure 27.

The different diffusion coefficients detected for these two molecules discarded the possibility of self or cross dimerization. The triplet pattern was detected for the NH peak of the molecule **26** with the separation of 13.03 Hz between adjacent peaks. This triplet collapses into a singlet in ^1^H{^19^F} experiment confirming the presence of coupling between ^1^H and ^19^F. This has been further ascertained by acquiring the spectrum in a high polarity solvent DMSO-*d*_6_ which resulted in the collapsing of triplet to a singlet confirming that this coupling is mediated through HB.

**Figure 28:**
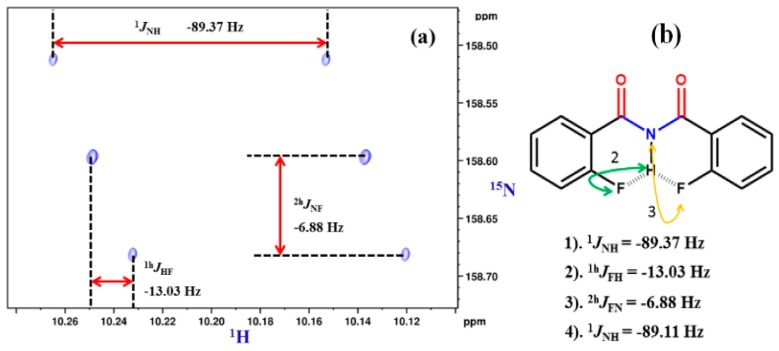

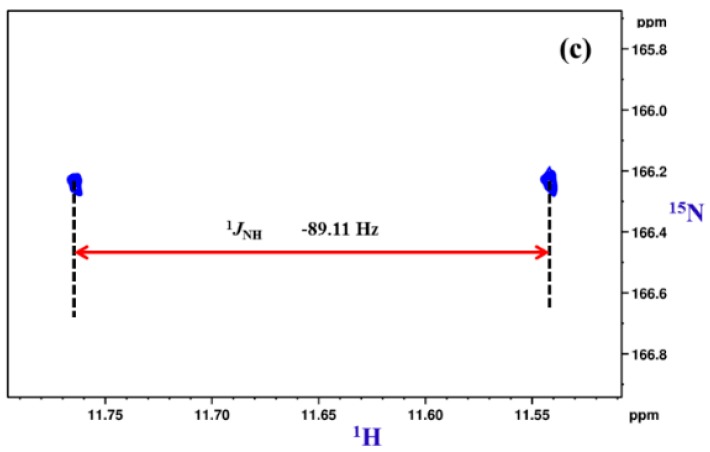
800 MHz spectrum of molecule **26** in CDCl_3_ (**a**) ^1^H-^15^N-HSQC (NH-coupled) spectrum; (**b**) the chemical structure of molecule **1** and measured couplings with their signs; (**c**) 400 MHz ^1^H-^15^N-HSQC spectrum (NH-coupled) in DMSO-*d*_6_.

The visualization of coupling became more prominent in the 2D ^1^H-^15^N HSQC NMR experiment and the corresponding spectrum for molecule **26** is reported in Figure 28. The ^1^H-^15^N HSQC spectrum of the same molecule in the solvent DMSO-*d*_6_ is reported in Figure 28c. In the solvent DMSO, except for ^1^*J*_NH_, all the other couplings disappeared, giving strong and unambiguous evidence that the measured couplings ^1h^*J*_FH_ and ^2h^*J*_FN_ in the solvent CDCl_3_ are mediated through HB. The ^1^H-^15^N HSQC spectra of all the other fluorine containing investigated molecules also exhibited through space couplings of different magnitudes.

**Figure 29:**
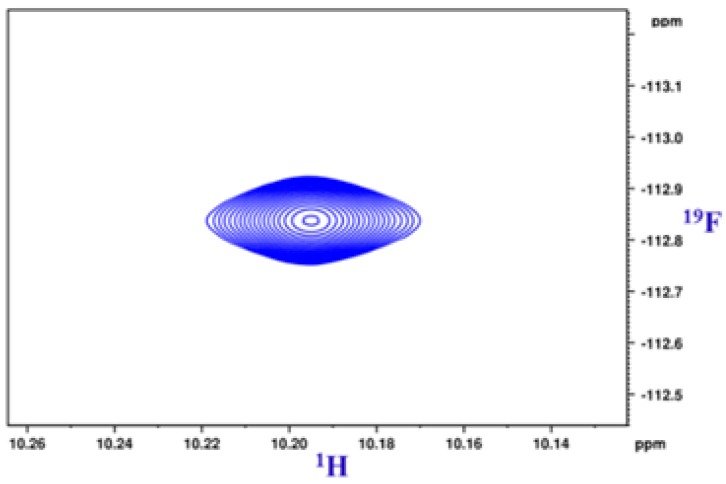
376.5 MHz 2D ^1^H-^19^F HOESY spectrum of the molecule **26** in CDCl_3_.

The ^1^*J*_NH_ values of the molecules **26**, **27** and **29**–**32** are substantially smaller than the molecule **28**, providing strong and unambiguous evidence that the nature of HBs in the derivatives of imides is predominantly of the covalent type [143,144] The strong correlations in the 2D ^1^H-^19^F HOESY [163,164,165] experiments for the molecules **26** and **29**–**32** established the close proximity between NH and F atoms in these molecules. The 2D ^1^H-^19^F HOESY spectrum of the molecule **26** is reported in the Figure 29.

### 5.2. Theoretical Calculations

The DFT [135,136] optimized structure calculations have been carried out to ascertain the NMR observations. The DFT calculations have been performed with B3LYP/6-311+g (d,p) level of theory with the chloroform as the solvation medium. The energy minimized structures were confirmed by harmonic vibrational frequency. The wave function files for QTAIM, and NCI studies and simulated ^1^H-NMR spectra using CSGT [145] were generated from optimized coordinates.

#### 5.2.1. Conformational Study

**Figure 30:**
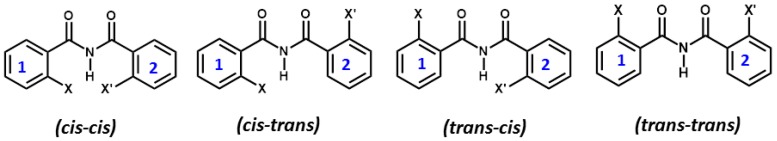
The possible conformers of the investigated imide molecules, arising due to the ring flip.

For all the investigated imides maximum of 3–4 major conformations are possible due to ring flip as shown in the Figure 30.

Out of the four possible conformers the cis-cis and trans-trans conformers were optimized and the difference {(*cis-cis*) – (*trans-trans*)} of minimum energy has been taken. The energy difference ΔE_cis-trans_ varied from approximately −2 to −11 Kcal/mol among all the investigated imide molecules and it is also found that the energy of *cis-cis* conformers is always lower than the *trans-trans* that are due to the presence of HB.

##### Relaxed Potential Energy Scan

**Figure 31:**
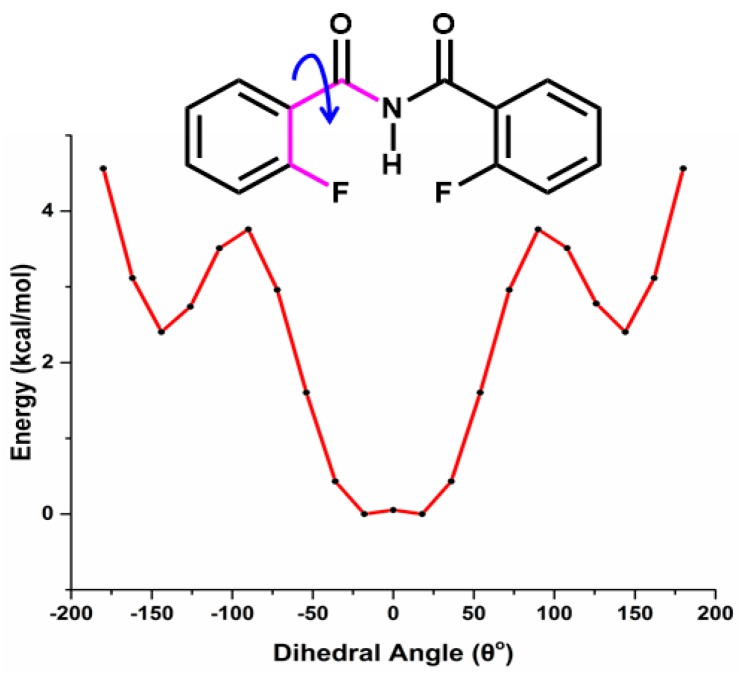
Relaxed potential energy surface for the internal rotation of the phenyl ring through single bond. The dihedral angle and rotation direction are highlighted in the structure of molecule **26**.

The findings of *cis-cis* and *trans-trans* energies of the conformers has been further supported by relax potential energy scans. Relaxed potential energy surface for the internal rotation of the phenyl ring through single bond has been performed. The rotation of 360° (−180–0–+180) through single bond was scanned in 20 steps with 18° of rotational segments. The scanned graph of energy (kcal/mol) versus dihedral angle (θ°) is reported in Figure 31. From the graph, it is clear that the energy of the *cis* conformer is lower than the other, and the maximum is for the *trans* conformation.

#### 5.2.2. Non Covalent Interaction (NCI) Calculations

The grid points based on NCI [90] calculations have been plotted for a defined real space function, sign(λ2_(r)_)ρ_(r)_, as function 1 and reduced density gradient (RDG) as function 2 and color filed isosurfaces are plotted for the investigated molecules. The NCI studies provided the visual information about weak interactions and repulsions in the molecules [158].

#### 5.2.3. Atoms in Molecules (AIM) Calculations

**Figure 32:**
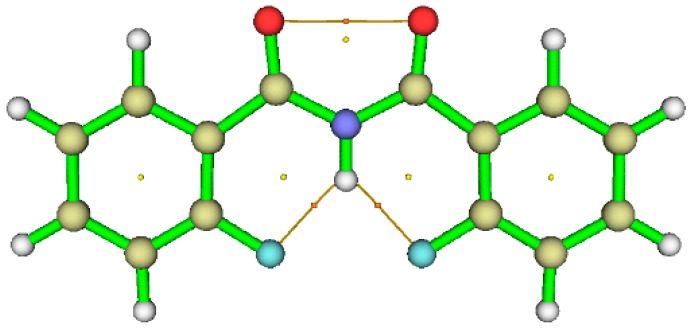
The visualization of BCPs and bond paths of HB for the molecule **26** plotted using the Multiwfn software. Dots represent the CPs and thin bar represents the HB interactions.

The magnitudes of ρ_(r)_, signs of ∇^2^ρ_(r)_ and potential energy density (*V*_(r)_) at corresponding (3, −1) BCP (bond critical points) (r_cp_) for HBs of interest have been calculated using QTAIM calculations [91,92,93,94,95]. The value of (*V*_(r)_) used in the *E*_HB_ = *V*(r_bcp_)/2 [96] for the calculation of energy of HB (*E*_HB_) of X∙∙∙HX type. The calculated *E*_HB_ of different HBs in all the investigated molecules varied from −2 to −8.56 Kcal/mol [158]. The bond paths for (3, −1) BCPs were generated for the investigated molecules for visualization and the molecular model for the molecule **26** containing BCPs and bond path is reported in Figure 32.

## 6. Studies on Diphenyloxamides

**Figure 33:**
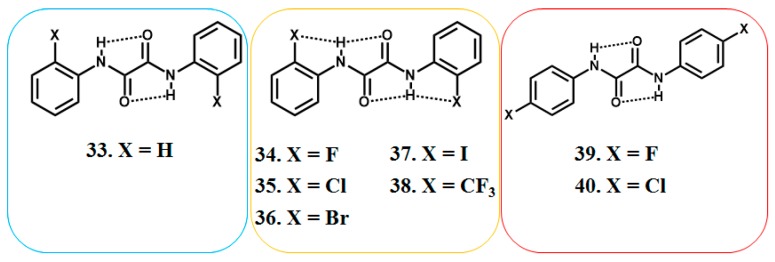
Chemical structures of *N,N*-diphenyloxamide (**33**) and its derivatives **34**–**40**. The dotted line3 indicate the HBs.

The existence of three centered C=O^…^H(N)^…^X-C HB involving organic fluorine, and other halogens in the derivatives of diphenyloxamide has been explored by NMR spectroscopy and quantum theoretical studies. The diphenyloxamide derivatives are the basic units of foldamers, where the formation of three centered HB contributes to the stable and rigid structure. The possible mode of three-centered HB formation C=O^…^H(N)^…^X-C (where X = CF_3_, F, Cl, Br and I) in the investigated molecules, along with their chemical structures are given in Figure 33.

### 6.1. Experimental Observation by NMR Spectroscopy

**Figure 34:**
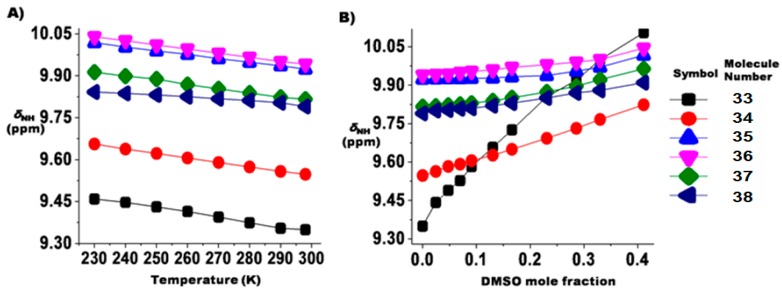
The variation of the chemical shift of NH proton (**A**) with temperature; (**B**) with the incremental addition of DMSO-*d*_6_ to the solution containing 200 µL of CDCl_3_. The 10 mM concentration at 298 K for the molecules, **33**–**38** has been initially taken for both the studies.

Initial studies were focused on the concentration dependence of NH chemical shift (δ_NH_) for all the molecules and it was observed that δ_NH_ remained unaltered for all the molecules, discarding the presence of any type of intermolecular interactions [166]. Compared to the unsubstituted molecule **33** the chemical shifts of NH protons are observed to be more deshielded for the different substituted molecules, **34**–**38**, indicating the participation of NH proton in weak molecular interactions [166]. The deshielding of NH chemical shift on lowering the temperature due to the strengthening in the HB is evident from Figure 34A.

The high polarity solvent DMSO-*d*_6_ induced perturbation of δ_NH_ is reported in Figure 34B. The larger value of Δδ_NH_/ΔV_DMSO_ in molecule **33** compared to the molecules, **34**–**38**, is giving an indication that the probable three centered H-bond is relatively stronger than the two centered one.

**Figure 35:**
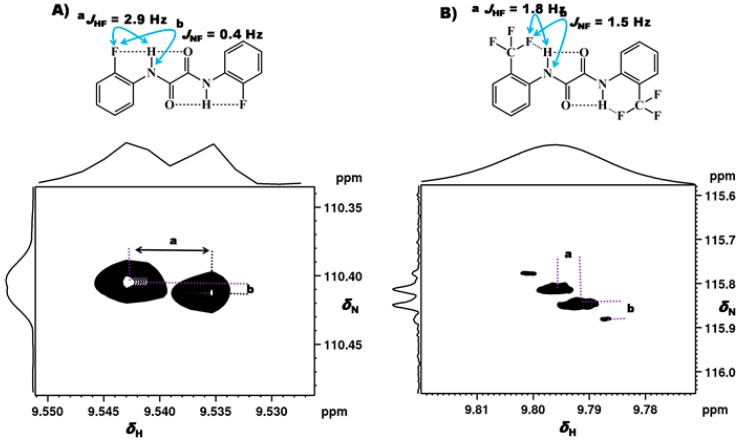
(**A**,**B**) ^15^N-^1^H-HSQC spectra of the molecules **34** and **38** respectively, in CDCl_3_. The molecular structures and the measured couplings have also been reported.

The 2D ^15^N-^1^H HSQC experiments for all the investigated molecules have been carried out, where ^15^N is in natural abundance. The ^15^N-^1^H HSQC spectrum of molecule **38** in CDCl_3_ yielded a quartet for the NH proton (reported in Figure 35B) implying that the rotation of CF_3_ group is very fast unlike in the earlier reported studies [79]. The ^15^N-^1^H HSQC spectrum of molecule **34** in the solvent CDCl_3_, reported in Figure 35A, yielded a doublet for the NH proton.

**Figure 36:**
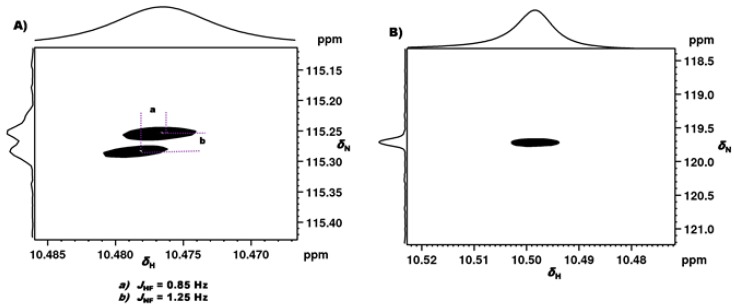
(**A**,**B**) ^15^N-^1^H HSQC spectra of the molecules **34** and **38** respectively, in DMSO-*d*_6_.

The quartet in ^15^N-^1^H HSQC spectrum of molecule **38** which was observed in the solvent CDCl_3_ collapsed to the singlet in the solvent DMSO-*d*_6_ reported in Figure 36B. This unambiguously established the existence of HB in the molecule **38**.

The molecule **34** in solvent DMSO-*d*_6_ yielded a doublet as reported in Figure 36A, with the *J*_HF_ = 0.85 Hz and *J*_NF_ = 1.25 Hz, whereas in CDCl_3_ these values were *J*_HF_ = 2.9 Hz and *J*_NF_ = 0.4 Hz, respectively. But it is observed that the relative slopes of the displacement vectors of the cross sections in the solvents (DMSO-*d*_6_ or CDCl_3_) are opposite (Figure 35A and Figure 36A). As the HB gets ruptured in the high polar solvents, the couplings observed in the DMSO-*d*_6_ are considered as through bond couplings and the observed coupling in the solvent CDCl_3_ is considered as through space coupling. Hence the contribution from the HB alone turns out to be −3.75 Hz.

As far as signs of *J*_NF_ is concerned, it has to be negative. These observations gave a strong evidence for the presence of HB in the molecule **34**. The close proximity between fluorine atom and NH protons has also been confirmed by the detection of cross peak in the 2D ^1^H-^19^F HOESY spectra of molecules **34** and **38**. This is additional support for the presence of intramolecular HB between F and NH proton [166]. An increase in the ^1^*J*_NH_ relative to molecule **33**, is observed in molecules **34**–**36** and **38**, indicating that the HB in these molecules is predominantly an electrostatic in nature [143,144]. On the other hand, ^1^*J*_NH_ decreases in molecule **37**, hence the nature of HB is predominantly covalent in this molecule [40].

### 6.2. Theoretical Studies

The observed experimental results have been also corroborated by DFT based theoretical calculations, such as, QTAIM [91,92,93,94,95], Natural Bond Orbital (NBO) [159] and NCI [90].

#### 6.2.1. QTAIM Calculations

**Figure 37:**
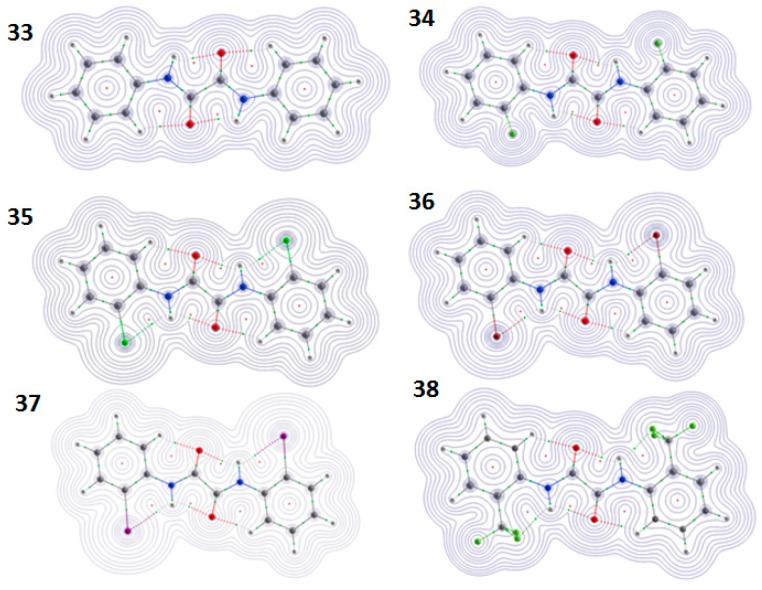
Molecular graphs, contour plots and AIM calculated bond paths for the molecules **33**–**38**. Dotted lines indicate the intramolecular HBs. Green and red dots are bond critical points (BCP) and ring critical points (RCP), respectively. Electron density is plotted as contours in the blue curves in the plane of molecules.

The magnitude of electron density (ρ) and sign of Laplacian of electron density (∇^2^ρ) are examined for the determination of HB. For such a purpose BCPs and bond paths for intramolecular HBs are detected for the investigated molecules and are reported in Figure 37.

The observed electron density values fall in the expected range (0.0102–0.0642 a.u.) for HBs [167]. The positive sign of Laplacian of electron density (∇^2^ρ) at the (3, −1) BCPs indicates the type of interactions that belong to the HBs. On the basis of electron density, the proton acceptance trend for organic halogens in the intramolecular five membered N-H^...^X follows the pattern Br > Cl > I, as detected from the chemical shift difference between the molecules **34**–**37** [166] compared to that of molecule **33**. This trend is further supported by the ρ(NH^...^X) values [166]. The obtained electron density values are about 0.015, 0.020 and 0.017 for NH^...^X, NH^...^O and CH^...^O, respectively. By using these values in the bonding energy (BE) equation; {BE (kj/mol) = 777 × ρ (in a.u.) − 0.4}, the BEs are estimated to be around 11, 15 and 13 kJ for NH^...^X, NH^...^O and CH^...^O HB interactions, respectively.

#### 6.2.2. NBO Analysis

For HB formation, electron transfer from the electron rich region of HB acceptor to the anti-bonding orbital (σ*) of the HB donor takes place. This has been studied by the Natural Bond Orbital (NBO) analysis and found that the values of lp(O) → σ*(N-H) were always greater than the lp(O) → σ*(C-H) values. Hence the N-H^…^O bonds are concluded to be stronger than the C-H^…^O bonds.

#### 6.2.3. NCI Analysis

**Figure 38:**
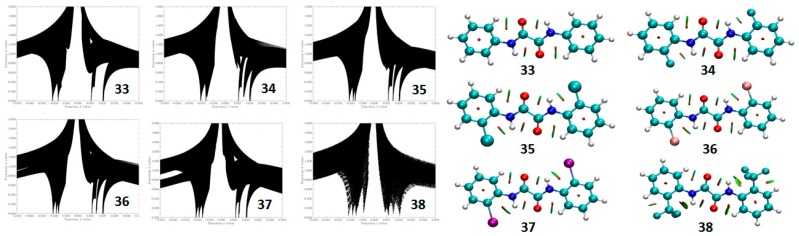
The plot of function 1 (sign(λ_2_)*ρ values) on X-axis vs function 2, the reduced density gradient (RDG) on *Y*-axis. (left hand side), colored isosurface plots in which Green colour denotes weak HB and red color stands for steric effect. Labels 33–38 represent molecules **33**–**38**, respectively.

QTAIM calculations and NBO analysis did not show the intramolecular BCP for N-H^…^F interaction in molecule **2** which contradicts the NMR observations. Another powerful method, NCI index has been used to visualize the weak interaction which showed the presence of HB in molecule **34**, which is in agreement with NMR observations. In Figure 38 (left side), the calculated grid points are plotted for the two functions: sign(λ_2_)*ρ as function 1 and reduced density gradient (RDG) as function 2 by Multiwfn program. Color filled isosurface graphs are plotted using these grid points and are reported in the right side of the Figure 38.

For the molecules **35**, **36**, and **37**, there are three spikes on the left-hand side which denote three HBs namely N-H^…^X, N-H^…^O and C-H^…^O (where X = Cl, Br, and I), these three bonds can be also seen in the colored isosurface plots reported on the right side of the Figure 38. For X = F, all three bonds exist and the spikes corresponds to two HBs are overlapped, these three HBs also are clearly seen in the isosurfaces plot 2. For X = CF_3_, the four spikes have been seen that are also visible in isosurfaces plot 6.

#### 6.2.4. Relaxed Potential Energy Scan

**Figure 39:**
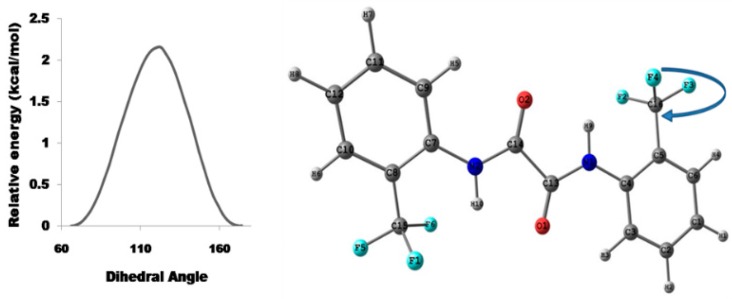
Relaxed potential energy surface for the internal rotation of CF_3_ group at B3LYP/6-311G** level of calculation. The dihedral angle H9–C5–C16–F2 has been selected for the scan.

It may be pointed out that in the ^15^N-^1^H-HSQC NMR spectrum of molecule **38** the NH group appeared as a quartet due to fast rotation of CF_3_ group at the ambient temperature. Relaxed potential energy scan for the H9–C5–C16–F2 dihedral angle was performed to derive the information about the energy barrier of internal rotation of CF_3_ group. This is reported in the Figure 39.

The barrier for this internal rotation was calculated as 2.15 kcal/mol using B3LYP/6-311G** level of theory and the energy barriers that can be observed in NMR is in between the 7–24 kcal/mol. This was the reason for observation of quartet for NH group.

## 7. Utility of H/D Exchange for Study of HB

The presence of HBs affects the release rate of labile hydrogen(s) which is/are participating in the reaction. There are number of chemical reactions in which the transfer of one or more protons (viz., hydride ions or hydrogen atoms) takes place in the rate determining step [40,42,43,44,45] The reaction kinetics depend on several factors, which also include the strength of intramolecular HB and the electronic effects caused by the substituents. The NMR spectroscopy is found to be a powerful technique which is widely employed for understanding the protein conformation, and dynamics [46,47,48,49,50,51,52] in aqueous media can be utilized to monitored by hydrogen/deuterium (H/D) exchange.

### 7.1. Factors Affecting the H/D Exchange

The rate of H/D exchange in a particular molecule can be affected by the inter- and the intra- molecular HBs. There will be a rapid exchange of the proton attached to nitrogen with the labile protons of the solvent. The mechanism of H/D exchange has been reported earlier in the derivatives of amides [53]. In the non-deuterated solvents, the proton exchange does not reflect in the NMR spectrum. On the other hand, in the presence of a labile deuterium-containing solvent the exchange takes place with the deuterium of the solvent molecules. This will have significant effect on the time dependent variation in the NMR signals intensities and permits the determination of H/D exchange rate. If the deuterated solvent is used in excess compared to the substrate, then the rate of exchange follows the pseudo first order kinetics. It is well known that the rate of H/D exchange is not only dependent on the strength of the intramolecular hydrogen bond, but also dependent on the electronic effect of the substituents.

**Figure 40:**
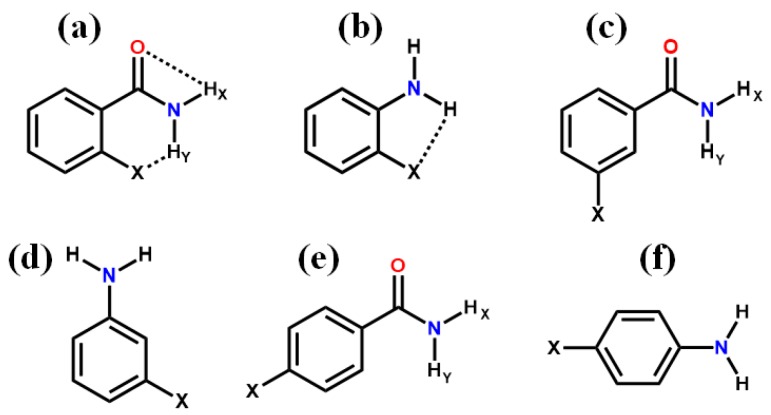
The general chemical structures of investigated halo substituted benzamides and anilines. For (**a**) and (**b**) X = F, Cl, Br, I and H and for (**c**), (**d**), (**e**), and (**f**) X = F, Cl and H.

The amides and amines are the basic building blocks for the synthesis of heterocyclic and linear nitrogen containing compounds. Consequent to significant difference in electronegativity (EN) and size among halogens, the strength of hydrogen bonds, steric hindrance and electronic effects [168] by these groups would also be substantially different. Hence the electronic effect, size effect and the effect of organic fluorine involved intramolecular HB on H/D exchange using ^1^H-NMR spectroscopic techniques has also been explored in the different halo substituted anilines and benzamides [169]. The chemical structures of all the investigated molecules are reported in Figure 40.

For all investigated molecules the 5 mM stock solution was prepared on the volume scale of 5 mL in the fresh CDCl_3_ solvent and the ^1^H-NMR spectra have been obtained using 450 µL of this stock solution. To this solution 50 µL of CD_3_OD was added with the marking time as zero, resulting in the final substrate concentration of less than 5 mM. This was well below the concentrations where the possibility of any aggregation and intermolecular interaction is observable. The added methanol created a 10% methanol:chloroform solution, with the final methanol concentration of 2.47 M, which ensured pseudo-first-order kinetics. The NMR spectra were acquired at every two minutes’ interval until the amino proton signal intensity submerged within the baseline noise. A distinct non-exchangeable aromatic proton peak was used as an internal integration reference. Rate constants and corresponding half-lives were determined from the slope of a nonlinear least squares fit to the graph of A(t) = A(o) exp^(^^−^^kt)^, rather than estimating the values at the extended time. The absolute intensity of Y intercept was taken 1 at zero time, and this value was used to normalize with respect to the remaining hydrogen. The X-intercept was the time. To ensure reproducibility the experiments have been repeated on a different sample at different time.

### 7.2. Exchange Mechanism

**Figure 41:**
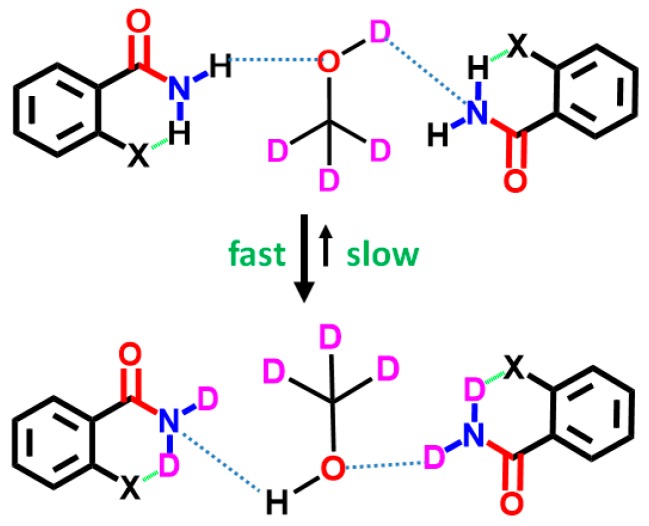
The mechanism of H/D exchange of labile protons with the hydroxy cdeuterium of the solvent molecule. The rate of deuteration is fast due to the high concentration of CD_3_OD compared to substrate.

On addition of CD_3_OD the labile protons undergo exchange with the hydroxy deuterium of the solvent molecule. This time dependent phenomenon followed the first order reaction kinetics and the intensity of the labile protons was systematically reduced. The mechanism of exchange is pictorially illustrated in Figure 41.

**Figure 42:**
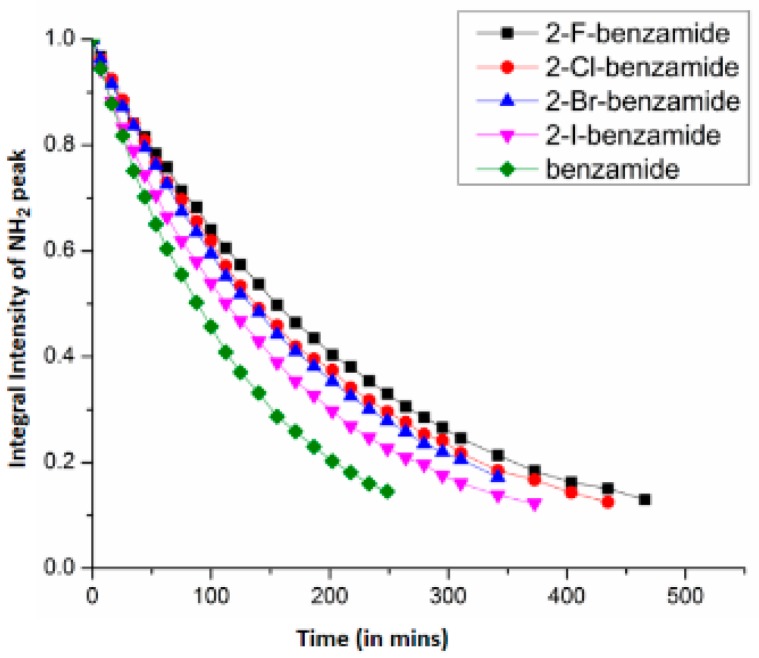
The plot of integral intensities of the NH_2_ peaks v/s time (in mins) for unsubstituted and ortho-halosubstituted benzamides. The molecules are identified by the symbols given in inset.

To get qualitative information about the strength of intramolecular HBs, and electronic effect the different halo substituted benzamide molecules were also investigated. The intensity of the peak at zero time is taken as 100%. The other spectra were acquired after adding 50 µL of CD_3_OD to 450 µL solution. The plot of integral areas of NH_2_ peaks as a function of time, for unsubstituted and *ortho*-halosubstituted benzamides (2-fluoro, 2-chloro, 2-bromo, 2-iodo) are compared in Figure 42. Similar results for *meta*- and *para*-substituted derivatives have also been obtained [169].

**Figure 43:**
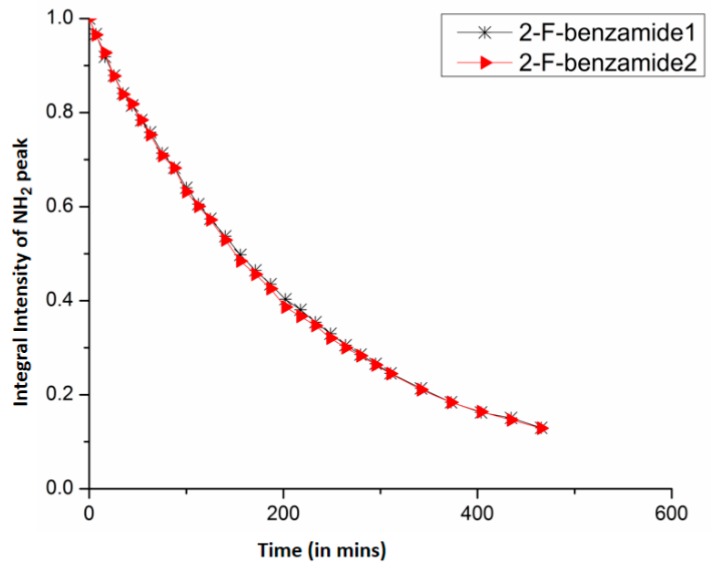
The integral intensity of NH_2_ peaks v/s time (in minutes) for 2-fluorobenzamide plotted from two experiments carried out at different times intervals are represented by two different symbols, shown in the inset.

The nature of the substituents dictated the H/D rate constants and they were different for different substituents. It has been observed that when all the physical parameters were kept unchanged for any of the investigated molecules, the results could be reproduced with high degree of accuracy within the experimental error [169]. There is a perfect match of graphical plots obtained from integral intensity of the labile protons peaks as a function of time, carried out for different samples at different times for 2-fluorobenzamide. This is clearly evident from Figure 43.

## 8. Multiple Quantum (MQ) NMR for the Detection of Intramolecular HBs

In deriving the evidence for C-F^…^H-N HB it is very important to know the precise magnitudes and also the relative signs of ^1h^*J*_FH_, ^3h^*J*_FH_, ^2h^*J*_FN_ and ^1^*J*_NH_ [65,69,170]. When three or more NMR active spins are coupled among themselves the magnitudes and signs of the couplings between the passive spins cannot be determined from the one-dimensional spectrum of any one of the coupled nuclei. Consequently, to obtain ^2h^*J*_FN_ in organofluorine molecules several experimental strategies have been reported where ^15^N is either isotopically labelled [111,170,171] or unlabelled [69].

### 8.1. The Pulse Sequence

**Figure 44:**
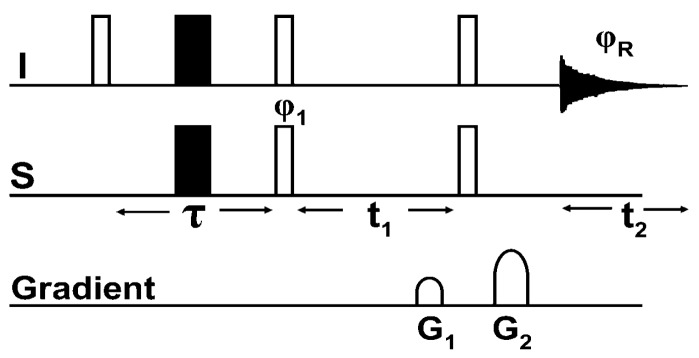
The pulse Sequence for DQ-SQ and ZQ-SQ experiments. For DQ and ZQ excitation, I and S spins are ^1^H and ^15^N of NH group. And the pulses were selective on these two spins. The phases of the pulses φ_1_ and φ_R_ are x, −x, −x, x. The phase of all the remaining pulses are x. The gradients ratio G_1_:G_2_ employed are 50:55 for DQ-SQ and 50:45 for ZQ-SQ experiments. The τ delay for all the DQ-SQ and ZQ-SQ experiments and also ^15^N-^1^H HSQC experiments was optimized for 1/2*J*_NH_ (*J*_NH_ is taken to be 90 Hz).

The utilization of two dimensional ^15^N-^1^H correlation (HSQC) type experiments [172,173] gives information on the magnitudes of ^1h^*J*_FH_, ^3h^*J*_FH_, ^2h^*J*_FN_ and ^1^*J*_NH_ but fails to provide the signs of the passive couplings. Hence the multiple quantum NMR experimental methodology has been employed [174,175]. The application of two dimensional heteronuclear ^15^N-^1^H double quantum-single quantum (DQ-SQ) and zero quantum-single quantum (ZQ-SQ) correlation experiment, where ^15^N is present in its natural abundance has been exploited for the determination of relative signs and magnitudes of the couplings among ^1^H, ^19^F and ^15^N, involved in hydrogen bonding [169]. The pulse sequence utilized for DQ-SQ and ZQ-SQ experiments is well known and is reported in the Figure 44.

**Figure 45:**
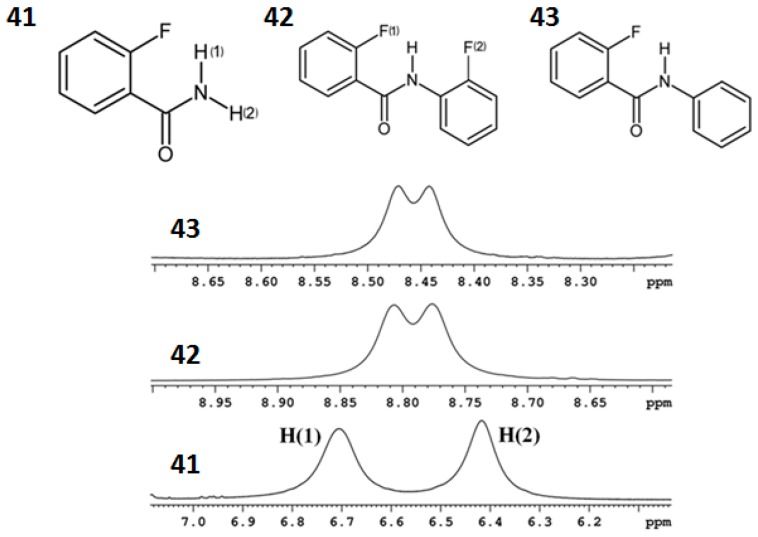
Chemical structures and expanded NH regions of the 1D ^1^H-NMR spectra in CDCl_3_ of 2-fluorobenzamide (**41**) 2-fluoro-*N*-(2-fluorophenyl)benzamide (**42**) and 2-fluoro-*N*-phenyl-benzamide (**43**).

The MQ experiments have been carried out on different fluorine substituted derivatives of benzamide in solution state in a low polarity solvent CDCl_3_. The magnitudes and signs of through space and long range coupling interactions among fluorine, hydrogen and nitrogen nuclei in its natural abundance have been used to extract direct evidence for the existence of non-linear and/or three centered intra-molecular C-F^...^H-N type HB. The chemical structures and 1D ^1^H-NMR spectra obtained for the investigated molecules are given in the Figure 45.

Subsequently the theoretically derived results [63] have been employed in conjunction with NMR findings to draw the direct and unambiguous conclusions on the intra-molecular C-F^…^H-N type HB.

### 8.2. C-F^…^N-H Hydrogen Bond in 2-Fluorobenzamide

**Figure 46:**
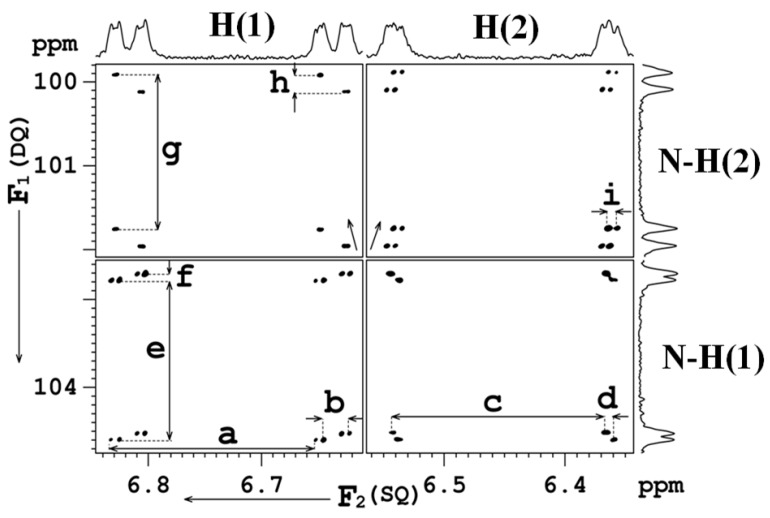
500 MHz ^15^N-^1^H DQ-SQ spectrum of molecule **41** in the solvent CDCl_3_.

The two broad singlets with a separation of nearly 145 Hz observed in ^1^H-NMR spectrum of molecule **41**, are attributed to two non-equivalent protons (δ_NH(1)_ > δ_NH(2)_) [111]. The excessive broadening of the ^1^H spectrum due to quadrupolar ^14^N relaxation prevented the determination of through space and through bond couplings, if any. For visualization of such couplings if present, the heteronuclear ^15^N-^1^H DQ-SQ correlation experiments have been carried out using the pulse sequence given in Figure 36 [176,177]. The ^15^N-^1^H DQ-SQ correlated spectrum is reported in Figure 37, where DQ coherence evolve at the algebraic sum of the couplings between active (^1^H, ^15^N) and passive (^19^F) spins. Nearly equal ^1^*J*_NH_ of each amide proton permitted the simultaneous excitation and detection of two different ^15^N-^1^H DQ spectra and are identified by the groups marked N-H(1) and N-H(2) in Figure 46.

**Figure 47:**
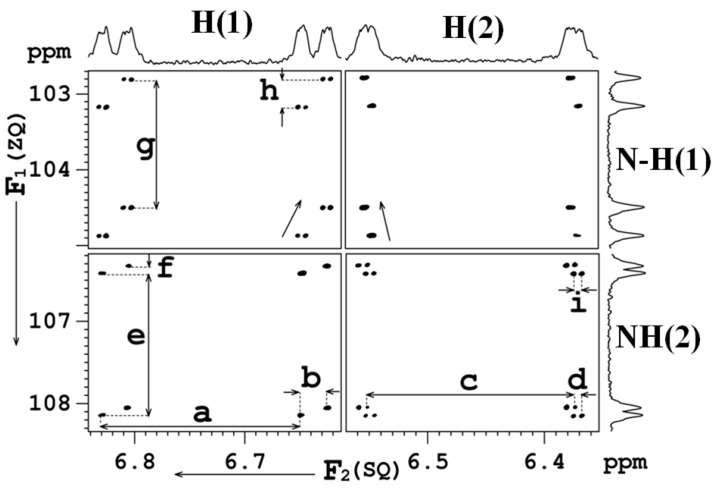
500 MHz ^15^N-^1^H ZQ-SQ spectrum of **41** in CDCl_3_.

For N-H(1) DQ excitation, H(1) and ^15^N are the active spins and H(2) and ^19^F are passive spins, while for N-H(2) DQ, H(2) and ^15^N are the active spins and H(1) and ^19^F are passive spins. The SQ dimension yields the normal spectrum of four coupled spins, viz., ^15^N, ^19^F and two NH protons. The nuclei involved in the DQ coherence flip simultaneously and can be treated as a single spin, also called as super spin [178]. The ^19^F and ^1^H being passive spins, the four possible spin states yield four transitions. The ^15^N-^1^H(1) DQ coherence gives the parameters ^1h^*J*_FH(1)_, ^2h^*J*_FN_, ^2^*J*_H(1)H(2)_ and ^1^*J*_NH(2)_ while ^15^N-^1^H(2) DQ coherence yields ^3h^*J*_FH(2)_, ^2h^*J*_FN_, ^2^*J*_H(1)H(2)_ and ^1^*J*_NH(1)_. The marked separations in the SQ dimensions of Figure 37 yield couplings (in Hz); a = ^1^*J*_NH(1)_ (90.5), c = ^1^*J*_NH(2)_ (89.2) and i = ^2^*J*_H(1)H(2)_ (3.05 Hz). The separations in DQ dimension provide magnitudes of the couplings (in Hz), g = ^1^*J*_NH(1)_ + ^2^*J*_H(1)H(2)_ (93.5), e = ^1^*J*_NH(2)_ + ^2^*J*_H(1)H(2)_ (92.2), f = ^1h^*J*_FH(1)_ + ^2h^*J*_FN_ (3.8) and h = ^3h^*J*_FH(2)_ + ^2h^*J*_FN_ (10.4). The displacement of F_2_ cross sections yield respectively (in Hz), b = ^1h^*J*_FH(1)_ (11.2) and d = ^3h^*J*_FH(2)_ (3.0). Opposite directions of F_1_ displacement vectors at δ_H(1)_ and δ_H(2)_, denoted by tilted arrows, confirm opposite signs of ^1h^*J*_FH(1)_ and ^3h^*J*_FH(2)_ [177,179]. The signs of ^1^*J*_NH(1)_ and ^1^*J*_NH(2)_ are negative because of the convention that one bond scalar coupling between two nuclei of opposite signs of magnetic moments is negative. The algebraic combination of parameters obtained from the DQ spectrum established that the relative signs of ^1h^*J*_FH(1)_ and ^3h^*J*_FH(2)_ are opposite, that of ^1h^*J*_FH(1)_, ^2h^*J*_FN_ and ^1^*J*_NH_ are same (negative), ^2^*J*_H(1)H(2)_ is positive. Thus, the magnitudes and relative signs of the couplings among all the three nuclei could be obtained from a single 2D NMR experiment. This information can be utilized for ascertaining the existence of C-F^…^H-N hydrogen bonding, if any, utilizing the theoretical results [63]. Nevertheless, for unequivocally ascertaining the relative signs of ^1h^*J*_FH(1)_, ^3h^*J*_FH(2)_ and ^2h^*J*_FN_, the ^15^N-^1^H zero quantum ^15^N-^1^H ZQ-SQ correlation experiment has also been carried out using the identical pulse sequence with appropriate gradient ratio and phases of the pulses. The ZQ coherence evolves at the algebraic difference of the couplings between active (^1^H, ^15^N) and passive (^19^F) spins. Consequent to the opposite signs of magnetic moments of excited spins the coherence evolved as algebraic sums [180]. This resulted in the enhanced separation along the ZQ dimension of ZQ-SQ spectrum given in Figure 47.

It is evident that signs of ^1h^*J*_FH(1)_ and ^2h^*J*_FN_ are opposite to ^3h^*J*_FH(2)_. The similar studies have also been carried out for other molecules and the obtained results established the presence of fluorine involved intramolecular HB.

## 9. Conclusions

The combined NMR experimental observations and various DFT based theoretical calculations revealed the existence of intramolecular HBs in different organofluorine-substituted derivatives of different classes of molecules, viz. benzanilides, hydrazides, imides, benzamides, and diphenyloxamides. The existence of more than one conformer has also been established in several molecules by adopting diverse NMR experimental strategies. In many examples the solvent titration and ^1^H DOSY techniques are employed to discount any possibility of self- or cross-dimerization. The variable temperature and solvent titration studies provided the valuable information about the existence of HB, and their relative strengths. The ^1^H-^19^F HOESY experiment is utilized to extract the information about the spatial proximity and possibility of intramolecular HB, as well as for the determination of different conformers. Through space coupling between two NMR active nuclei, where the spin polarization is transmitted through HB of diverse strengths has been detected in several fluorine containing molecules. The 2D ^1^H-^15^N HSQC experiment aided in the measurement of coupling strengths and their relative signs. The weak molecular interactions established by NMR studies have also been corroborated by theoretical DFT based structure calculations. The NCI analysis served as a very sensitive tool for the detection of non-covalent interactions. NCI analysis provides the visual evidence for the presence of bi and trifurcated HBs in some of the molecules. For the calculation of strengths of different HBs in the investigated molecules the QTAIM calculation is found to be very powerful tool. The Laplacian of electron density sign is used to discriminate the HBs from the covalent bonds. The relaxed potential energy scan has been performed for some of the molecules to determine the free rotation energy of CF_3_ group and the energies of different possible conformers for a molecule. The quantum calculations and classical MD simulations have supported the various possible ways of intramolecular HB formation with fluorine atom. The use of H/D exchange rates obtained by conventional ^1^H-NMR spectroscopic studies confirms the presence or absence of intramolecular HBs, and permitted the calculation of their relative strengths. The effect of inherent electronic and steric effects is also successfully correlated with the relative rates of H/D exchange in the various halo substituted derivatives of anilines and benzamides. The utilization of heteronuclear ^15^N-^1^H DQ-SQ and ZQ-SQ correlation experiment, in isotopically unlabeled systems for the detection of organic fluorine involved intramolecular HB and unequivocal determination of relative signs and magnitudes of both hydrogen bond and covalent bond mediated through scalar couplings such as ^1h^*J*_FH_, ^2h^*J*_FN_ and ^3h^*J*_FH_ has been convincingly demonstrated.

## 10. Computational Methods and the Employed Programs

For DFT calculations Gaussian G09 [181] suite of programs has been used. The B3LYP/6-311G** and aug-cc-pVTZ level of theories and basis sets were used to optimize the structures of the molecules. To ensure that all optimized structures are global minima, harmonic vibrational frequency calculation has been performed at the same level of theories and all the real frequencies indicated that optimized geometry is minimum at potential energy surface. The MD simulations were performed using NAMD simulation package [182]. The general AMBER force field (GAFF) along with torsion parameters and partial charges obtained from quantum calculations in vacuum. The long range electrostatic interactions were calculated with the Particle Mesh Ewald (PME) method [183]. Constant pressure-temperature (NPT) simulation is performed followed by constant volume-temperature (NVT) simulation. AIMAll [184] and multiwfn programs were used for QTAIM calculations. Multiwfn [185] program was used for NCI plots. Colour filled isosurface graphs have been plotted by VMD [186] program. Specific combination of methods for theoretical calculations was used for particular and individual series of molecules, the specific information is available with the native articles. Molden 4.7 has been used as a visualization software for Gaussian outputs [187].
