# Correction: Mishra, S.K. and Suryaprakash, N. Intramolecular Hydrogen Bonding Involving Organic Fluorine: NMR Investigations Corroborated by DFT-Based Theoretical Calculations: *Molecules* 2017, *22*, 423

**DOI:** 10.3390/molecules24102000

**Published:** 2019-05-24

**Authors:** Sandeep Kumar Mishra, N. Suryaprakash

**Affiliations:** NMR Research Centre, Solid State and Structural Chemistry Unit, Indian Institute of Science, Bangalore 560012, India; sand2epmishra@gmail.com

The authors wish to make the following corrections to their paper [1]. The authors regret that in the above-mentioned paper, Figure 2 and the associated text were adapted without attribution from “Molecular Interactions (Noncovalent Interactions) and the Behaviors of Biological Macromolecules” by Loren Dean Williams [2]. The authors sincerely thank Prof. Loren Dean Williams for bringing this to our attention.

## Figures and Tables

**Figure 2:**
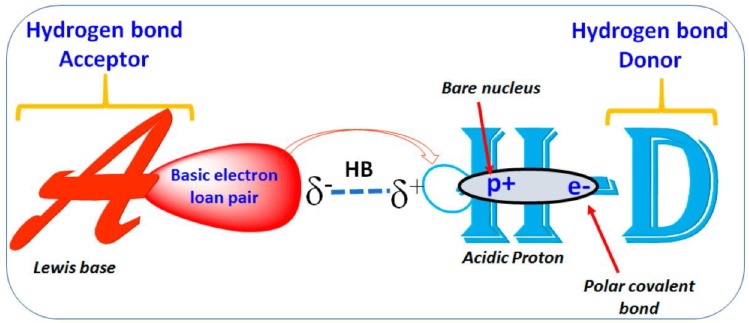
The pictorial illustration of hydrogen bond interaction, where HB acceptor/donor can be an F, O, N, or S atom in the molecule. Electron polarization and exposure of a positive proton on either side are shown schematically.
